# Cellular Characterization and Interspecies Evolution of the Tree Shrew Retina across Postnatal Lifespan

**DOI:** 10.34133/research.0536

**Published:** 2024-11-21

**Authors:** Liu-Lin Xiong, Yi-Fei Sun, Rui-Ze Niu, Lu-Lu Xue, Li Chen, Li-Ren Huangfu, Jing Li, Yu-Ying Wang, Xin Liu, Wen-Yuan Wang, Zhong-Fu Zuo, Ting-Hua Wang

**Affiliations:** ^1^ Department of Anesthesiology, Research Institute of Neurosurgery, West China Hospital, Sichuan University, Chengdu 610041, Sichuan, China.; ^2^ Department of Anesthesiology, The Third Affiliated Hospital of Zunyi Medical University, Zunyi 563000, Guizhou, China.; ^3^ Department of Urology, the Second Affiliated Hospital of Kunming Medical University, Kunming 650500, China.; ^4^ Mental Health Center of Kunming Medical University, Kunming 650034, Yunnan, China.; ^5^ State Key Lab of Biotherapy, West China Hospital, Sichuan University, Chengdu 610041, Sichuan, China.; ^6^ Institute of Neuroscience, Kunming Medical University, Kunming 650500, Yunnan, China.; ^7^ Department of Anatomy, College of Basic Medicine, Jinzhou Medical University, Jinzhou 121001, Liaoning, China.; ^8^ Interdisciplinary Research Center on Biology and Chemistry, Shanghai Institute of Organic Chemistry, Chinese Academy of Science, Shanghai 200032, China.

## Abstract

Tree shrews (TSs) possess a highly developed visual system. Here, we establish an age-related single-cell RNA sequencing atlas of retina cells from 15 TSs, covering 6 major retina cell classes and 3 glial cell types. An age effect is observed on the cell subset composition and gene expression pattern. We then verify the cell subtypes and identify specific markers in the TS retina including *CA10* for bipolar cells, *MEGF11* for H1 horizontal cells, and *SLIT2*, *RUNX1*, *FOXP2*, and *SPP1* for retinal ganglion cell subpopulations. The cross-species analysis elucidates the cell type-specific transcriptional programs, different cell compositions, and cell communications. The comparisons also reveal that TS cones and subclasses of bipolar and amacrine cells exhibit the closest relationship with humans and macaques. Our results suggests that TS could be used as a better disease model to understand age-dependent cellular and genetic mechanisms of the retina, particularly for the retinal diseases associated with cones.

## Introduction

Primates have long been considered the most suitable animal models for studying human diseases. However, research using primates is limited mainly due to their high costs and time consumption, and stringent requirements for laboratory animals and scientific research facilities. The tree shrew (*Tupaia belangeri*; TS) is an animal species with a unique evolution of dominant organs and distinct evolutionary relationships between primates and rodents [1,2]. Being one of the closest living relatives of primates (*Eeuarchontogliers*), TS possesses a cone-dominant retina with short-wave sensitivity (SWS) and long-wave sensitivity (LWS) cones [3], and its highly developed visual system shares various features with primates [4]. RNA sequencing analysis revealed that TS shares high homology with humans in terms of Alzheimer’s disease (AD)-related differentially expressed genes (DEGs) in human brain tissues [5]. Additionally, TS have been employed in vision studies [6–8], as they are a diurnal mammalian species closely related to primates that have multiple advantages for the translational study of visual prosthetics. TS retina is primarily composed of cones [9], which is advantageous for prosthetic vision research as humans typically operate in illuminated environments. Additionally, as a diurnal mammal that heavily relies on vision [10], the TS can be easily trained on visually based cognitive behavioral tasks [7,11]. Therefore, it is essential to explore whether TS can serve as a suitable animal model for studying age-related retinal disorders.

The retina is a special photosensitive tissue composed of various cell types, including 5 major classes of retinal cells: photoreceptors (rods and cones); retinal ganglion cells (RGCs), which are classically considered neurons due to their ability to generate action potentials; horizontal cells (HCs); bipolar cells (BCs); amacrine cells (ACs); and non-neuronal Müller glial cells [12]. Photoreceptors in the outer nuclear layer (ONL) sense light and transmit visually evoked signals to interneurons (HCs, BCs, and ACs) in the inner nuclear layer (INL); then, the interneurons process the information that is supplied to RGCs in the innermost layer; and the RGCs finally send axons through the optic nerve to cerebral visual centers [13]. As development progresses and age increases, the photoreceptor structure of the retina degrades, especially in the fovea area of primates, accompanied by a decrease in the proportions of photoreceptors and a decline in visual processing ability [14]. Retinal cell populations vary in composition and proportion according to the age of humans, primates, and rodents [15]. It is essential to elucidate the entirety of the complexity of the retina at an individual cell-type resolution across the postnatal lifespan in TS. In addition, the evolutionary characteristics of the retina that exhibit consistency in different species remains to be elucidated.

Single-nucleus RNA sequencing (snRNA-seq) is highly beneficial for obtaining the transcriptome at the individual cell level in the retina, which can offer critical insights into age-related retinal biology and disease [16]. Recently, snRNA-seq studies have been performed on the retinas of different species, including mice [17], macaques [18], chicks [13], and humans [19]. These studies have provided critical high-resolution transcriptomic data for conventional retinal cell types and allowed the discovery of novel cell subtypes in different animals. In this study, we profiled 107,718 single nuclei from retinal samples of 15 healthy TS donors during postnatal lifespan using the snRNA-seq approach, of which the age range fulfilled the criteria based on the previous publication [20]): infant (1.4 to 2.1 months), mature (24.96 to 36 months), and senile (85.8 to 86.56 months). We identified 9 cell populations with different gene expression characteristics, including cones, rods, BCs, HCs, ACs, RGCs, Müller, astrocytes, and microglia. Cell type-specific transcriptional alterations, specific cell markers, molecular localization, and dysregulated cell-to-cell communication in the TS retina were comprehensively elucidated. A cross-species analysis was also employed to depict the underlying similarities or differences of the single-nucleus transcriptomic landscape, cell composition, and cell communications among TS, humans, macaques, mice, and chicks. Collectively, we provide a retinal age-dependent cellular and single-cell genetic atlas of TS retina, validating the further use of TS for modeling retinal development and disorders.

## Results

### Cell-type taxonomy of the TS retina at different age stages

**Fig. 1.**
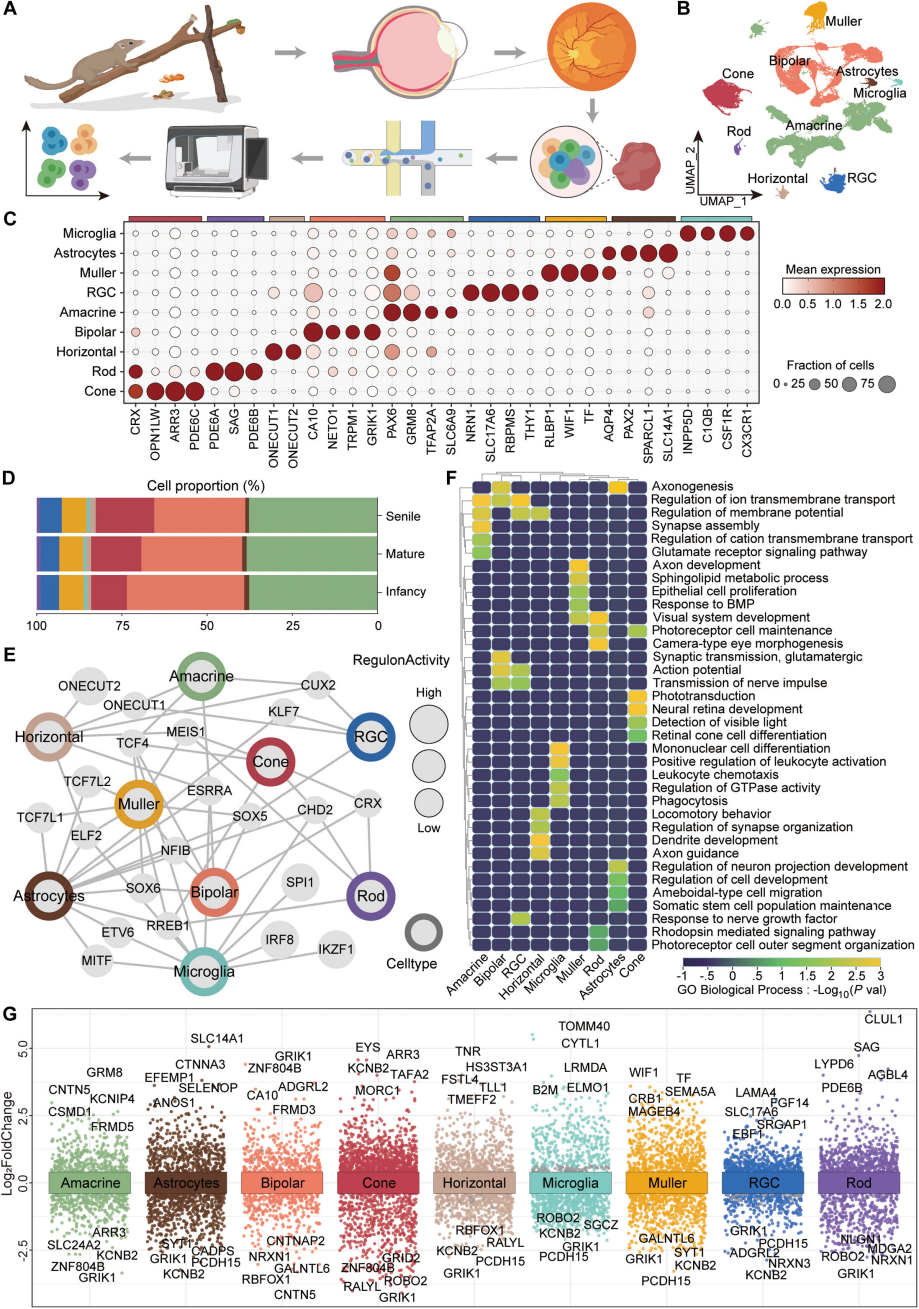
Cellular composition of retinas in the infant, mature, and senile TS delineated by single-nucleus transcriptome profiles. (A) snRNA-seq experimental workflow of this study. *n* = 5 TSs per group. (B) UMAP map shows the distribution of all cell clusters in TS retina. (C) Dot plot shows expression levels and distribution of representative cell markers across 9 cell populations. The size of the dot represents the percentage of cells expressed by the selected genes in a cluster. The degree of color refers to the average expression of genes within cells. (D) The proportions of 9 major cell types in infant, mature, and senile TS groups. (E) The network map exhibits the specific transcriptional regulators of 9 cell types in the TS retina. (F) The heatmap provides the representative GO terms enriched by DEGs of each cell type. (G) Differential gene expression analysis shows DEGs in each cell type, and the top 5 up-regulated or down-regulated DEGs are marked. UMAP, uniform manifold and projection; RGC, retinal ganglion cell.

To comprehensively elucidate the cellular composition and molecular signatures of the TS retina during development, we isolated cells from 15 retinas of infant, mature, and senile subjects, which were subjected to a 10× Genomics platform for snRNA-seq (Fig. 1A and Fig. S1A). After strict quality control, 107,718 cells including 38,578 cells from infant TS, 35,436 cells from mature TS, and 33,704 cells from senile TS were used for downstream analysis (Fig. S1B and Table S1). Next, 29 cell clusters were confirmed after unbiased clustering using uniform manifold and projection (UMAP), of which the cell number was displayed (Fig. S1C and D). Upon unsupervised clustering, we divided all cells into 9 cell types according to the specific gene characteristics expressed in each cluster (Fig. 1B and C and Table S1), including 6 types of retinal cells (cones, *CRX*^+^, *OPN1LW*^+^, *ARR3*^+^, *PDE6C*^+^; rods, *CRX*^+^, *PDE6A*^+^, *PDE6B*^+^, *SAG*^+^; BCs, *CA10*^+^, *NETO1*^+^, *GRIK1*^+^, *TRPM1*^+^; HCs, *ONECUT1*^+^, *ONECUT2*^+^; ACs, *PAX6*^+^, *GRM8*^+^, *TFAP2A*^+^, *SLC6A9*^+^; RGCs, *RBPMS*^+^, *SLC17A6*^+^, *NRN1*^+^, *THY1*^+^) and 3 types of glia cells (Müller cells, *RLBP1*^+^, *TF*^+^, *WIF1*^+^; astrocytes, *PAX2*^+^, *SPARCL1*^+^, *AQP4*^+^, *SLC14A1*^+^; microglia, *INPP5D*^+^, *CSF1R*^+^, *C1QB*^+^, *CX3CR1*^+^) visualized using UMAP (Fig. 1C and Fig. S1E and F). Based on these attributes, cellular compositions in the TS retinas of different ages were revealed, showing an increasing trend in the number of RGCs and cones with age, while the proportion of BCs and the 3 non-neuronal cell types (Müller cells, microglia, and astrocytes) showed a decreasing trend with age (Fig. 1D and Fig. S2A).

Analysis of the transcription factors with high transcriptional activity for each cell type revealed unique transcriptional features and enriched biological processes relevant to their distinct physical functions (Fig. 1E and F and Table S1). For example, RGCs, Müller, astrocytes, and BCs shared the transcription factor *SOX5* (Fig. 1E), which is known to be involved in the regulation of cellular differentiation and the maintenance of specific cell fates [21], suggesting that this transcription factor may act as a common regulatory factor contributing to both neuronal and glial development and ensuring the proper formation and function of retinal circuits. RGCs, ACs, and HCs all transcribed *ONECUT1,* which is essential for HC genesis and retinal integrity [22], and HCs specifically expressed *ONECUT2* (Fig. 1E). Gene Ontology (GO) terms related to axon and retina development including “axonogenesis”, “axon development”, “synapse assembly”, “neural retina development”, “visual system development”, and “regulation of membrane potential” were enriched for BCs, Müller, ACs, cones, rods, and RGCs, respectively (Fig. 1F and Table S1). We performed differential gene expression analysis among different cell populations. DEGs in each cell type were visualized using a volcano plot. The top 5 up-regulated or down-regulated genes are depicted, including some marker genes seldom reported before, such as rods (*CLUL1*) and BCs (*CA10*) (Fig. 1G).

Then, we analyzed and compared the functional characteristics of the up-regulated DEGs in the infant and senile groups. The GO terms revealed that except for RGCs, the remaining 8 cell types were all involved in the “eye development” process at the infant stage, whereas “oxidative phosphorylation” (rods and amacrines), “netrin-activated signaling pathway” (cones), and “negative regulation of response to oxidative stress” (RGCs) were enriched in the senile group (Fig. S1G and H and Table S1). For cones, “eye development” and “visual perception” were prominently enriched in the infant group, and “regulation of neuron projection development” and “axonogenesis” were also enriched in the senile group (Fig. S1G and H and Table S1), indicating the potentially protective effect of cones against age-related retinal function decline in TS. These further demonstrated the positive regulation of related cell types in the retinal functions.

### Characteristics of photoreceptor cells and identification of specific cell markers in TS

**Fig. 2.**
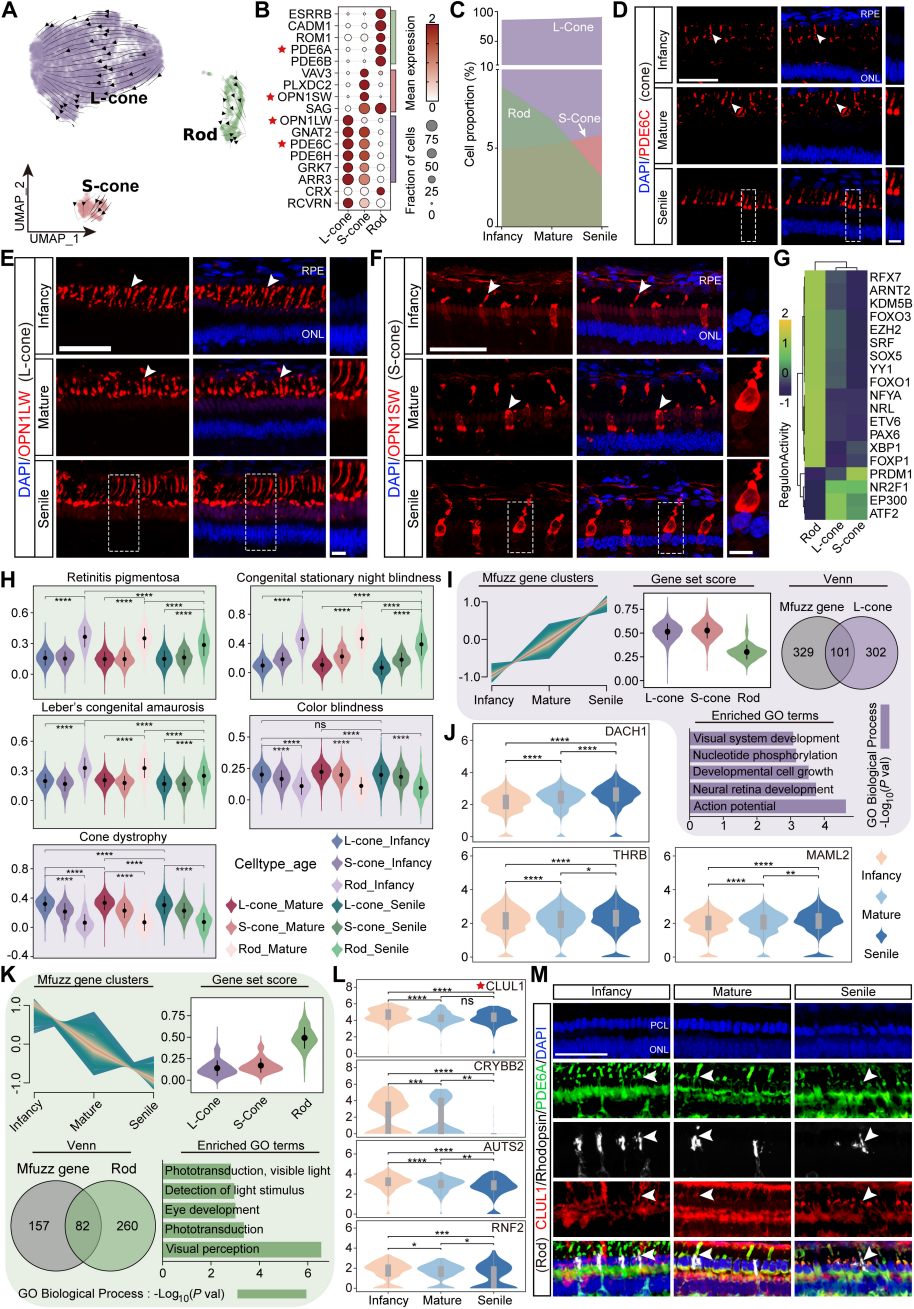
Classification and characterization of photoreceptor cells. (A) UMAP presentation of the 3 TS retinal photoreceptor cell subpopulations. (B) Expression of representative marker genes in 3 subpopulations. The size of the dot represents the percentage of cells expressed by the selected genes in a cluster. The degree of color refers to the average expression of genes within cells. (C) Proportional changes of 3 photoreceptor cell subpopulations across infant, mature, and senile. (D to F) Immunostaining identification of cone cells [*PDE6C*^+^, red, (D)] and 2 subpopulations, L-cone [*OPN1SW*^+^, red, (E)] and S-cone [*OPN1LW*^+^, red, (F)], in the infant, mature, and senile groups. Blue, DAPI. Scale bar: low magnification, 50 μm; high magnification, 10 μm. *n* = 3 TSs per group. (G) Transcription factor expression of the photoreceptor cell subpopulations. (H) Disease-related gene set scores of RP, CSNB, LCA, color blindness, and cone dystrophy. (I) Mfuzz plot clusters the up-regulated genes in photoreceptor cells with age. The association of these up-regulated genes with DEGs in the cones and rods was revealed by gene set scoring. Venn plot of age-related up-regulated genes in photoreceptor cells with up-DEGs in L-cone (adjusted *P* < 0.05, log_2_FC > 0.25). The GO terms enriched by the overlapped up-DEGs in L-cones. (J) The Violin diagram presents the expression of *DACH1*, *THRB*, and *MAML2* in the infant, mature, and senile groups. (K) Mfuzz plot clusters the down-regulated genes in photoreceptor cells with age. The association of these down-regulated genes with cones and rods was revealed by gene set scoring. Venn plot of age-related down-regulated genes in photoreceptor cells with down-DEGs in rods (adjusted *P* < 0.05, log_2_FC > 0.25). The GO terms enriched by the overlapped down-DEGs in rods. (L) Violin plots show the expression of *CLUL1*, *CRYBB2*, *AUTS2*, and *RNF2* in rod cells among infant, mature, and senile groups. (M) Triple immunostaining of *CLUL1*^+^ rods [*PDE6A*^+^ (green)/Rhodopsin^+^ (white)/*CLUL1*^+^ (red)] in the TS retina at the infant, mature, and senile groups. Scale bar, 50 μm. *n* = 3 TSs per group. Similar outcomes were obtained in 3 repeated independent experiments. White arrows indicate the positive immunostaining, and the white dashed boxes represent the zoom area of the positive immunostaining. *P ≤ 0.05, **P ≤ 0.01, ***P ≤ 0.001, and ****P ≤ 0.0001. ns, no significance; RPE, retinal pigment epithelium; ONL, outer nuclear layer; INL, inner nuclear layer; PCL, photoreceptor cell layer; L-cone, longwave-sensitive cones; S-cone, shortwave-sensitive cone cells.

TSs are dichromatic and exhibit 2 types of cone cells, namely, shortwave-sensitive cone cells (S-cones) expressing blue opsin (*OPN1SW*) and longwave-sensitive cones (L-cones) expressing red opsin (*OPN1LW*) [23]. After strict quality control (Materials and Methods), 15,381 photoreceptors were analyzed, of which 94% were cones and 6% were rods; they were clustered into 6 clusters with distinct gene expression (Fig. S3A and Table S2). According to the markers specifically expressed in each cluster, the photoreceptors cells were divided into L-cones (*OPN1LW*^+^, *GNAT2*^+^, *PDE6C*^+^, *PDE6H*^+^, *GRK7*^+^, *ARR3*^+^, *RCVRN*^+^, C0-C3), S-cones (*VAV3*^+^, *PLXDC2*^+^, *OPN1SW*^+^, *SAG*^+^, *GNAT2*^+^, *PDE6C*^+^, C5), and rods (*PDE6A*^+^, *PDE6B*^+^, *SAG*^+^, *CADM1*^+^, *ESRRB*^+^, *ROM1*^+^, *CRX*^+^, C4) (Fig. 2A and B and Fig. S3B). Overall, as age increased, the proportion of cones showed an increasing trend, while the proportion of rods exhibited a decreasing trend (Fig. 2C, Fig. S2B, and Table S2). The cones were distributed in the ONL and layer of rods and cones (RCL) in the TS retina, as indicated by *PDE6C*^+^ (cones), *OPN1LW*^+^ (L-cone), and *OPN1SW*^+^ (S-cone) immunostaining of TS retina (Fig. 2D to F). Moreover, cones and rods contained distinct cell-specific transcription factors (Fig. 2G). The disease gene set score showed that rods consistently show higher susceptibility scores for diseases such as retinitis pigmentosa (RP), congenital stationary night blindness (CSNB), and Leber’s congenital amaurosis (LCA) in infancy, maturity, and senility. Meanwhile, cones, especially in mature and senile stages, exhibit higher associations with cone dystrophy and color blindness (Fig. 2H). The prominently higher susceptibility of rods to RP, CSNB, and LCA, as compared to cones, particularly highlighted the vulnerability of rod cells to degeneration with age. Next, we explored age-related up-regulated genes in photoreceptor cells, and obtained 101 genes after intersecting with up-DEGs in L-cones, which were mainly involved in such a lot of eye-beneficial biological processes as “visual system development”, “nucleotide phosphorylation”, “developmental cell growth”, “neural retina development”, and “action potential” (Fig. 2I and Table S2). We identified 5 crucial genes (*DACH1*, *THRB*, *MAML2*, *INPP4B*, and *CADM2*) whose expression was consistently elevated with age in the L-cones (Fig. 2J and Fig. S3C). *DACH1* encodes a chromatin-associated protein that correlates with other DNA-binding transcription factors and is a key component in the regulation of cellular fate [24]. Thus, the augmentation of cones in the TS retina with increasing age may be attributed to genetic regulation. Regarding rods, the results revealed 82 intersected genes between the age-related down-regulated genes in photoreceptor cells and down-DEGs in rods, which also took participation in processes beneficial for vision including “phototransduction, visible light”, “detection of light stimulus”, “eye development”, “phototransduction”, and “visual perception” (Fig. 2K). The changes in the cell proportion of rod cells may be linked to decreased levels of these genes. Furthermore, as shown in Fig. 1I, *CLUL1* stood out as the most up-regulated DEGs in rod cells. Here, we found that the high expression levels observed in infant TS decreased in both mature and senile TS (Fig. 2L, Fig. S3D, and Table S2). In addition, *CRYBB2*, *AUTS2*, and *RNF2*, which are related to the retinal development, were also decreased in rods from infancy to senile (Fig. 2L). Immunostaining outcomes identified *CLUL1* expressing in rods with triple positivity for *PDE6A*^+^/Rhodopsin^+^/*CLUL1*^+^ (Fig. 2M). Here, our data presented different cell proportions of cones and rods with age and verified several specific cell markers of the cones (*OPN1SW, OPN1LW*) and rods (*CLUL1*) in the TS retina. Crucial DEGs in the developmental process were also revealed, which might underlie the mechanisms of age-related retinal morbidity.

### Cellular and molecular profiling of BCs in TS

**Fig. 3.**
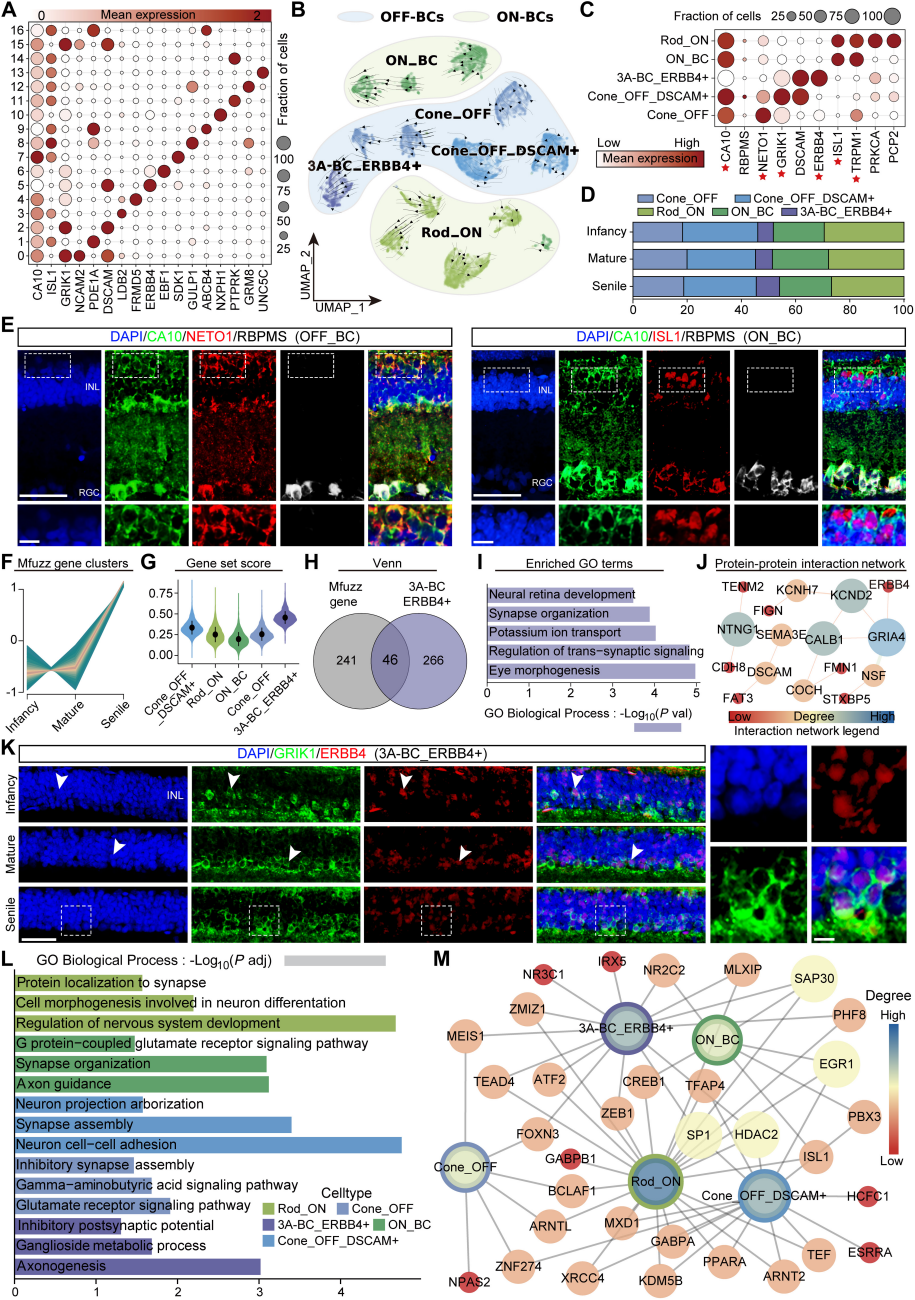
Classification and characterization of BCs and their subsets. (A) Expression of selected marker genes in 17 subclusters of BCs. (B) The distribution of annotated OFF_BCs and ON_BCs subpopulations is visualized using UMAP. (C) Expression of representative marker genes in 5 BC subpopulations. The size of the dot represents the percentage of cells expressed by the selected genes in a cluster. The degree of color refers to the average expression of genes within cells. The red 5-pointed stars represent important marker genes. (D) Proportions changes of 5 BC subpopulations in infant, mature, and senile groups. (E) Immunostaining identification of OFF_BCs [*CA10*^+^ (green)/*NETO1*^+^ (red)/*RBPMS*^−^ (white)] and ON-BCs [*CA10*^+^ (green)/*ISL1*^+^ (red)/*RBPMS*^−^ (white)] in the mature TS retinas. Scale bar: low magnification, 50 μm; high magnification, 10 μm. *n* = 3 TSs per group. (F) Mfuzz plot clusters the up-regulated genes in BCs with age. (G) Association of up-regulated genes with 5 BC subpopulations was revealed by gene set scoring. (H) Venn plot of age-related up-regulated genes in BCs with up-DEGs in *ERBB4*^+^ BCs (adjusted *P* < 0.05, log_2_FC > 0.25). (I) GO terms enriched by the overlapped up-DEGs in *ERBB4*^+^ BCs. (J) PPI network demonstrates interaction among these overlapped up-DEGs. (K) Double immunostaining of *GRIK1*^+^ (green)/*ERBB4*^+^ (red) in BCs in the infant, mature, and senile groups. Blue, DAPI. Scale bar: low magnification, 50 μm; high magnification, 10 μm. *n* = 3 TSs per group. Similar outcomes were obtained in 3 repeated independent experiments. White arrows indicate the positive immunostaining, and the white dashed boxes represent the zoom area of the positive immunostaining. (L) GO enrichment analysis of 5 BC subsets. The horizontal coordinator is −log_10_ (*P* adj). (M) The network diagram shows the transcription factor regulation of 5 BC subpopulations. GO, Gene Ontology.

We obtained 29,695 BCs based on the pan-bipolar marker *NETO1* combined with *TRPM1* and *GRIK1* and reclustered these cells into 17 clusters (Fig. S3E and Table S3). The results demonstrated that these 17 cell clusters simultaneously expressed *CA10* (Fig. 3A and Fig. S3E and F). In mammals, BCs are usually divided into 2 types based on their response to light: ON bipolar cells (ON_BCs), which primarily receive input from rods and depolarize in response to light, and OFF bipolar cells (OFF_BCs), which primarily receive input from cones and hyperpolarize in response to light [25]. The aforementioned 17 clusters were divided into OFF_BC (*GRIK1*^+^, C0, C2, C4 to C6, C10, and C15) and ON_BC (*ISL1*^+^, C1, C3, C7 to C9, C11 to C14, and C16) (Fig. S3E and F and Fig. 3B). ON_BCs consisted of Rod_ON and ON_BC subpopulations, while OFF_BCs were made up of Cone_OFF, Cone_OFF_*DSCAM* (abbreviated as *DSCAM*^+^), and 3A_BC_*ERBB4* (abbreviated as *ERBB4*^+^) subsets (Fig. 3B and C). The cell composition was exhibited at infant, mature, and senile TS retina, where ON_BCs increased in the mature group (*P* = 0.035 versus infancy) and Rod_ON decreased in the senile group (*P* = 0.019 vs infancy) (Fig. 3D and Fig. S2C). Interestingly, *CA10* was presented as a novel TS BC marker identified by the triple immunofluorescent staining of *CA10*^+^/*NETO1*^+^/*RBPMS*^−^ (OFF_BCs) and *CA10*^+^/*ISL1*^+^/*RBPMS*^−^ (ON_BCs) in the INL at the different age stages (Fig. 3E). Given the rising cellular proportional trend in *ERBB4*^+^ subpopulation, we searched for genes that showed increased expression during aging in BCs and intersected them with up-DEGs in *ERBB4*^+^ subset, by which 46 overlapped genes were obtained and their enriched biological processes included “neural retina development”, “synapse organization”, “regulation of trans-synaptic signaling”, and “eye morphogenesis” (Fig. 3F to I and Table S3). The protein–protein interaction (PPI) analysis also revealed the crucial regulation between *GRIA4* and *ERBB4* (Fig. 3J), indicating the important roles of ERBB4 in axon regulation [26,27]. We then identified *ERBB4*^+^ BCs (*GRIK1*^+^/*ERBB4*^+^) located in the INL layer of TS retina (Fig. 3K). It has been reported that the synergistic effect of *ERBB4* and neuregulin (*NRG*) further promotes important functions such as cell proliferation, neurite outgrowth, and myelination. Simultaneously, they also regulate neuronal excitability and synaptic plasticity, which play a crucial role in the nervous system [28]. Furthermore, GO analysis showed that the *ERBB4*^+^ subpopulation was mainly enriched in “inhibitory postsynaptic potential”, “ganglioside metabolic process”, and “axonogenesis” (Fig. 3L). In addition, the *ERBB4*^+^ cell population specifically expresses retinal cell development molecules such as *IRX5* [29], *MEIS1* [30], and *HDAC2* [31] and also expresses specific autophagy-related transcription factors like ATF2 [32] (Fig. 3M).

In summary, we identified a specific cell marker, *CA10*, for BCs in the TS retina. Moreover, the increased trend of *ERBB4*^+^ BCs proportion with age may be involved in promoting axon regeneration, resulting in resistance to retinal degradation with age in TS.

### Cell subpopulations of TS ACs

**Fig. 4.**
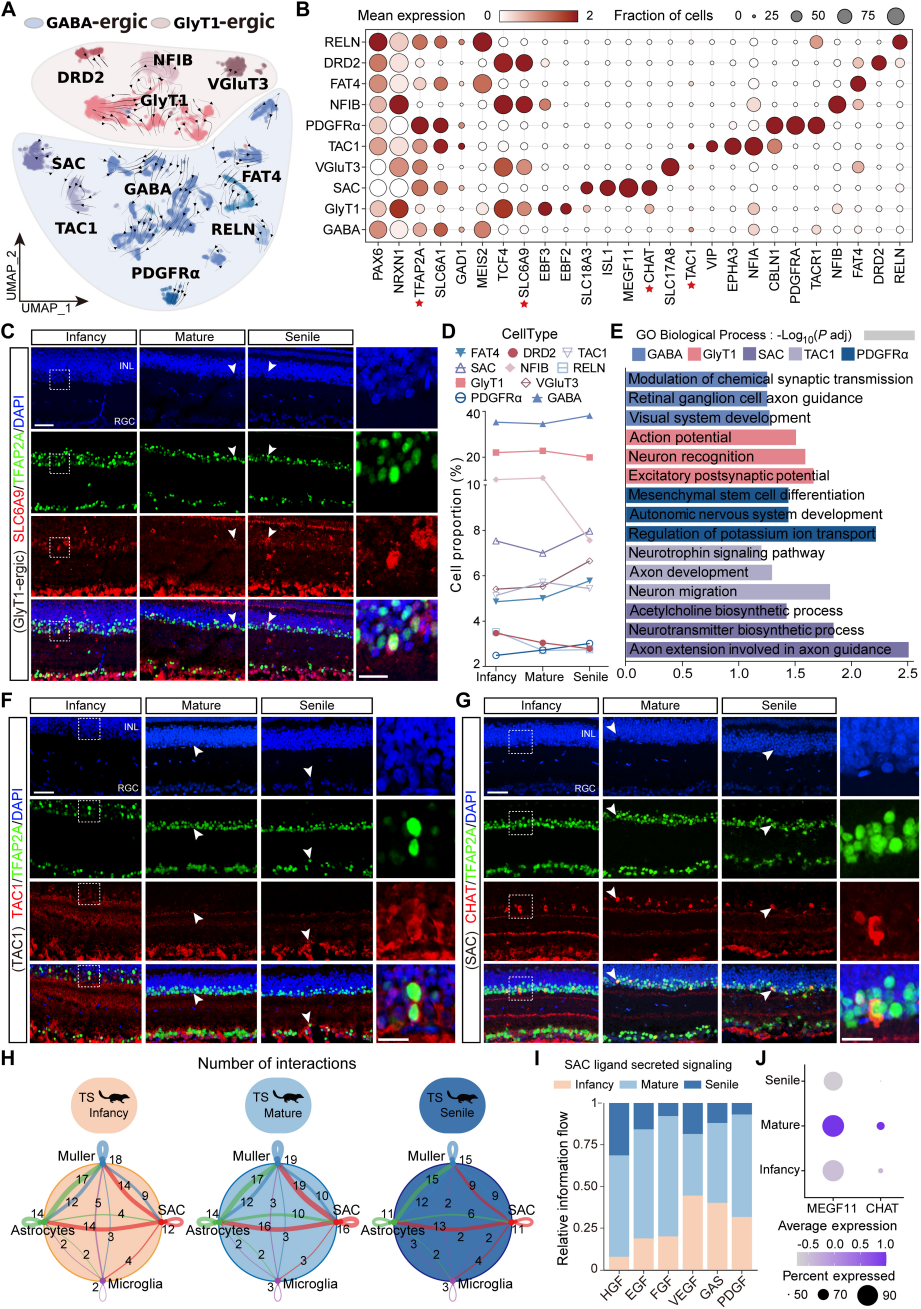
Classification and characteristics of ACs and subsets in TS retina. (A) AC subclusters are visualized using UMAP. (B) The dot plot presents the expression of the selected marker genes across 10 subclusters. The size of the dot represents the percentage of cells expressed by the selected genes in a cluster. The degree of color refers to the average expression of genes within cells. The red 5-pointed stars represent important marker genes. (C) Immunostaining identification of *GlyT1*^+^ ACs [*TFAP2A*^+^ (green)/*SLC6A9*^+^ (red)] in TS retina at different stages. Blue, DAPI. Scale bar: low magnification, 50 μm; high magnification, 25 μm. *n* = 3 TSs per group. (D) Proportions of 10 AC subsets among the infant, mature, and senile groups. (E) GO enrichment analysis of 5 AC subpopulations. The horizontal coordinator is −log_10_ (*P* adj). (F and G) Immunostaining identification of *TFAP2A*^+^ (green)/*TAC1*^+^ (red) and *TFAP2A*^+^ (green)/*CHAT*^+^ (red) ACs in the infant, mature, and senile groups. Blue, DAPI Scale bar: low magnification, 50 μm; high magnification, 25 μm. *n* = 3 TSs per group. Similar outcomes were obtained in 3 repeated independent experiments. White arrows indicate the positive immunostaining, and the white dashed boxes represent the zoom area of the positive immunostaining. (H) The network plot shows the relative information flow among SACs, astrocytes, microglia, and Müller cells. (I) The bar chart displays the number of ligand–receptor interactions of SACs at the infant, mature, and senile stages. (J) Expression of *CHAT* and *MEGF11* in SACs across the infant, mature, and senile groups. TS, tree shrews; SACs, starburst amacrine cells.

To identify the specific AC subtypes within the molecularly defined clusters, we reclustered 34,936 ACs and obtained 28 distinct clusters (Fig. S4A). Through transcriptional profiling, we identified genes uniquely expressed in each cluster (Fig. S4B and Table S4). Based on the markers *SLC6A1*^+^ and *TCF4*^+^, ACs were classified into 6 *GABA*-ergic and 4 *GlyT1*-ergic clusters. Among the *GABA*-ergic clusters, we identified subtypes such as starburst AC (*SAC*s, *CHAT*^+^), as well as subsets defined by markers such as *GABA^+^*, *FAT4^+^*, *TAC1^+^*, *RELN^+^*, and *PDGFRA^+^*. Similarly, *GlyT1*-ergic ACs consisted of *DRD2^+^*, *NFIB^+^*, *GlyT1^+^*, and *VGluT3^+^* subpopulations (Fig. 4A and B). Distribution of *GlyT1*-ergic ACs was verified in the INL and RGC layers of the TS retina by double immunofluorescent staining of *TFAP2A*^+^/*SLC6A9*^+^ (Fig. 4C). The cell proportion of AC subtypes was exhibited at different age stages (Fig. 4D, Fig. S2E, and Table S4). GO analysis revealed that AC subpopulations, including SACs, *TAC1*^+^, *PDGFA*^+^, *GlyT1*^+^, and *GABA*^+^ cells, were mainly involved in processes that enhance axon development and visual system development, such as “axon extension involved in axon guidance”, “axon development”, and “retinal ganglion cell axon guidance” (Fig. 4E and Table S4). *TFAP2A*^+^/*TAC1*^+^ and *TFAP2A*^+^/*CHAT*^+^ immunofluorescent staining demonstrated the location of *TAC1*^+^ and *CHAT*^+^ ACs in the INL and RGC layers of the TS retina (Fig. 4F and G). The communication of SACs with astrocytes and Müller was strengthened in the mature group but weakened in the senile stage with corresponding alternated ligand signaling, such as *FGF*, *PDGF*, and *VEGF* (Fig. 4H and I). In addition, the expression of cholinergic neuronal markers (*CHAT* and *MEGF11*) in SACs was consistent with the changes in cell communication intensity (Fig. 4J). In total, SACs, as the most specific subtype of TS ACs, might underlie the axon regulation in the retinal development.

### Characteristics and identification of the RGC subpopulations

**Fig. 5.**
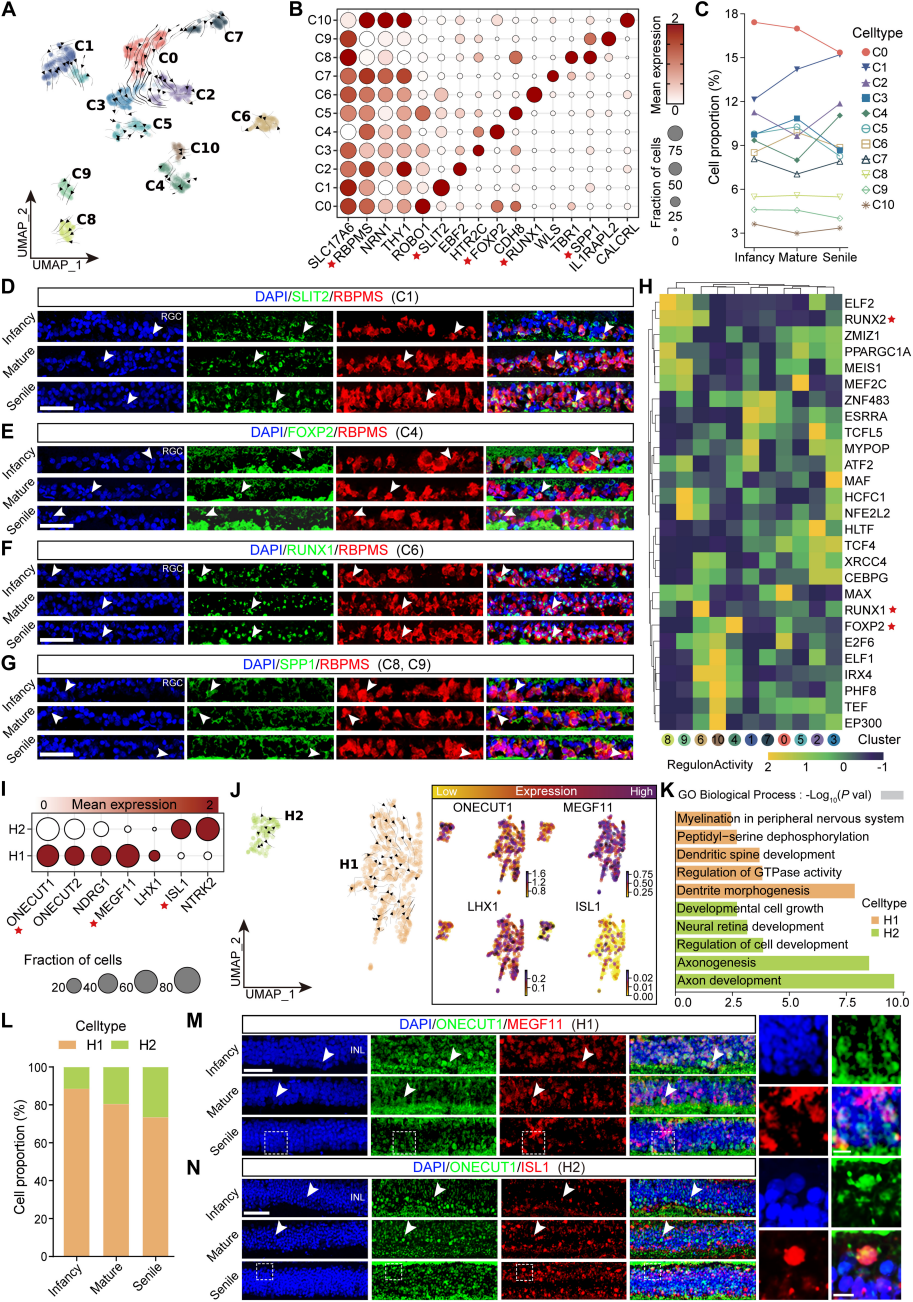
Features and identification of RGCs and HCs in the TS retina. (A) RGC subclusters are visualized using UMAP. (B) Expression of representative marker genes in RGC subclusters. The size of the dot represents the percentage of cells expressed by the selected genes in a cluster. The degree of color refers to the average expression of genes within cells. The red 5-pointed stars represent important marker genes. (C) Proportions of cellular subgroups in infant, mature, and senile groups. (D to G) Immunostaining identification of *SLIT2*^+^ (green)/*RBPMS*^+^ [red, (D)], *FOXP2*^+^ (green)/*RBPMS*^+^ [red, (E)], *RUNX1*^+^ (green)/*RBPMS*^+^ [red, (F)], and *SPP1*^+^ (green)/*RBPMS*^+^ [red (G)] RGCs in the infant, mature, and senile groups. Blue, DAPI. Scale bar: 50 μm. *n* = 3 TSs per group. (H) The network shows the regulatory relationship of transcriptional factors across RGC subclusters. (I) Expression of the representative marker genes of 2 HC subpopulations. The red 5-pointed stars represent important marker genes. (J) Distribution of HC subtypes are visualized using UMAP. (K) Annotation of GO terms enriched by DEGs in H1 and H2. The horizontal coordinator is −log_10_ (*P* val). (L) Proportions of HC subtypes in the infant, mature, and senile groups. (M and N) Immunostaining identification of H1 [*ONECUT1*^+^ (green)/*MEGF11*^+^ (red) (M)] and H2 [*ONECUT1*^+^ (green)/*ISL1*^+^ (red) (N)] in the infant, mature, and senile groups. Blue, DAPI. Scale bar: low magnification, 50 μm; high magnification, 10 μm. *n* = 3 TSs per group. Similar outcomes were obtained in 3 repeated independent experiments. White arrows indicate the positive immunostaining, and the white dashed boxes represent the zoom area of the positive immunostaining.

Marker characterization was performed to exclude RGCs that did not express pan-RGC markers (Fig. S4C and D and Table S1), and the remaining 5,593 RGCs were reclustered into 11 clusters, all expressing the typical pan-RGC markers *NRN1*, *RBPMS*, *SLC17A6*, and *THY1* (Fig. 5A and B and Fig. S4D). C6 selectively expresses *RUNX1* (Fig. 5B), a runt-associated transcription factor related to retinal angiogenesis, and has been identified as a marker for a subtype of RGCs [33,34]. C4 selectively expresses *FOXP2*, a transcription factor of the forkhead/winged-helix (FOX) family. Additionally, C1 specifically expressed *SLIT2* (Fig. 5B), which is an RGC growth cone collapse factor inhibiting RGC axon guidance [35]. C8 and C9 selectively express secreted phosphoprotein 1 [SPP1, also known as osteopontin (OPN), a marker for α-RGCs], which is a secreted glycosylated phosphoprotein that can affect cell survival, inflammation, migration, and homeostasis after injury [36]. Subsequently, the changes in the proportion of RGC subtypes were unveiled with advancing age, with *FOXP2*^+^ RGCs increased in the senile group (*P* = 0.018 versus mature) (Fig. 5C and Fig. S2F). The distribution of *SLIT2^+^*, *FOXP2^+^*, RUNX1^+^, and *SPP1^+^* RGCs had not been previously verified in the TS retina, which were uncovered in the RGC layer of the TS retina indicated by double immunostaining of *SLIT12^+^/RBPMS^+^*, *FOXP2^+^/RBPMS^+^*, *RUNX1^+^/RBPMS^+^*, and *SPP1^+^/RBPMS^+^*, respectively (Fig. 5D to G). Enrichment analysis showed that C0 (*ROBO1^+^*) and C1 (*SLIT2*^+^) contributed to axonogenesis and visual system development (Fig. S4E). In addition, RGC subtype-specific transcriptional profiles were revealed, partly corresponding to the cluster-specific marker genes, such as *FOXP2* for C4 and *RUNX1* for C6, with particular expressed *RUNX2* in C8 and C9 (Fig. 5H). We further used the glaucoma disease-associated gene set to score RGC subpopulations, which revealed a higher disease score associated with α-RGCs (C8 and C9) at different periods than that with other subpopulations (Fig. S4F and Table S5). These findings revealed the crucial cellular and genetic characteristics of RGCs in the TS retina.

### Characteristics of HC subtypes with specific markers

HCs play important roles in visual information processing by performing lateral inhibition and modulating the signaling between photoreceptors and other retinal cells. Their actions help to refine the visual signals and optimize the transmission of information from the retina to higher visual centers in the brain [12,37]. In the mouse retina, there is only one identified HC type, but the primate retina contains at least 2 HC distinct subtypes, in which H1 receives input from both rods and cones, while H2 receives input from cones [38]. Here, we also reclustered 1,150 HCs of the TS into H1 and H2 subtypes based on the expression of the LIM homeodomain protein *ISL1*^+^ (Fig. 5I and J, Fig. S4G, and Table S6). The H1 type (C0, C1) expressed the LIM homeodomain transcription factor (*LHX1*) and cell surface receptors *MEGF11*, which can regulate *LHX1*^+^ H1 cell chimerism and play a key role in HC mosaic formation [39]. The H2 type (C2) expressed *ISL1* (Fig. 5J). In addition, the cell ratio of H1 and H2 was exhibited from infancy to senile stages, and GO analysis revealed that H1 is closely related to the nervous system development like “dendrite morphogenesis”, “dendritic spine development”, and “peptidyl-serine dephosphorylation and myelination in the peripheral nervous system”, while H2 was devoted to retinal development processes such as “axon development”, “axogenesis”, and “neural retina development and developmental cell growth” (Fig. 5K and L, Fig. S2D, and Table S6). Thus, H1 and H2 may be indispensable for biological processes conducive to visual processing and perception [40–42]. Furthermore, immunostaining identified that H1 (*MEGF11*^+^) and H2 (*ILS1*^+^) HCs were located in the INL of the TS retina (Fig. 5M and N). Conclusively, *MEGF11* is identified as a specific H1 subtype marker in this study.

### Diversity of TS glial cells

First, 7,643 glial cells were clustered into 3 classes without bias (Fig. S5A), Müller cells (*RLBP1*^+^, *WIF1*^+^, *CHRDL1*^+^, *TF*^+^), astrocytes (*AQP4*^+^, *GFAP*^+^, *NTRK2*^+^, *SLC14A1*^+^), and microglia (*AIF1*^+^, *PTPRC*^+^, *CX3CR1*^+^, *CSF1R*^+^, *C1QB*^+^) (Fig. S5B and Table S7), which participate in the development of anatomical and functional connections between neurons and surrounding tissues, facilitate the transport of ions and nutrients, ensure homeostasis of the microenvironment, and provide nutritional support for neuronal activity in the retinal development and angiogenesis [43]. The proportions of Müller, astrocytes, and microglia cells were shown from infancy to senile stages (Fig. S5C and Table S7). The TooManyCells tree explained that Müller and astrocytes were in the same branch, and greater correlation between Müller and astrocytes was also uncovered by clumpiness and Sperman correlation analysis (Fig. S5D and E and Table S7). We then clustered the down-regulated genes in glial cells and intersected them with down-DEGs in microglia. As a result, 75 common DEGs were overlapped and mainly enriched in “Notch signaling pathway”, “amyloid precursor protein catabolic process”, and “regulation of leukocyte differentiation” (Fig. S5F). Similarly, 122 up-DEGs were obtained by intersecting up-regulated genes in glial cells with up-DEGs in Müller cells, which were primarily involved in “regulation of nervous system development”, “axonogenesis”, and “synapse organization” (Fig. S5F). Moreover, 68 down-DEGs intersected from down-regulated genes in glial cells and down-DEGs in astrocytes participated in “regulation of neuron projection development”, “neural crest cell differentiation”, “positive regulation of protein autophosphorylation”, “neuron projection guidance”, and “axon development” (Fig. S5F). Differential gene expression analysis showed top 10 up- and down-regulated genes across Müller, astrocytes, and microglia (Fig. S5G). The immunostaining results showed the location of Müller cells (*WIF1*^+^) in the INL and RGC layers, astrocytes (*GFAP*^+^) in the RGC layer, and a wide distribution of microglia in all layers of the TS retina (Fig. S5H to J).

### Cross-species comparison of retinal cellular composition and transcriptomes

**Fig. 6.**
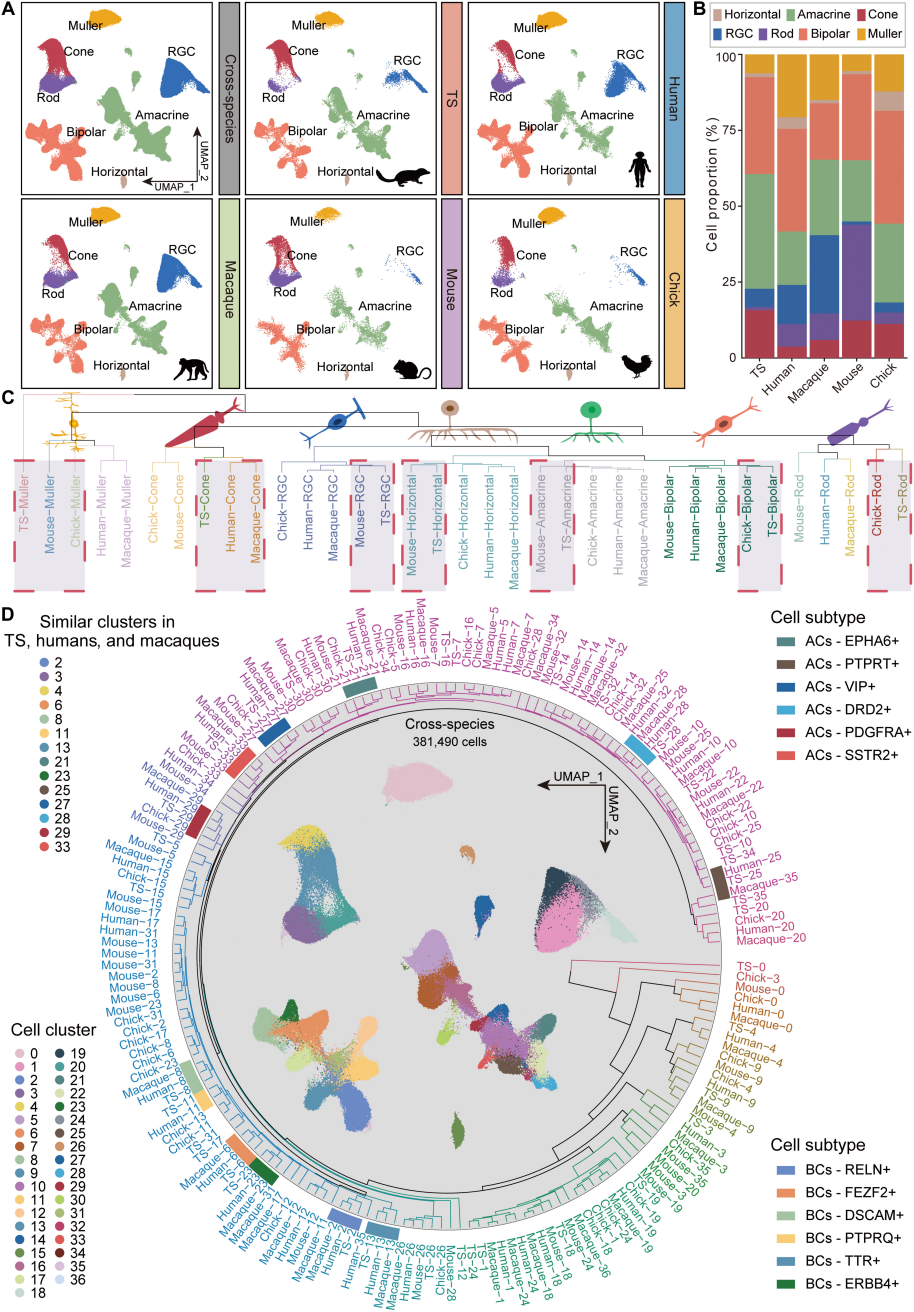
Cross-species comparison of retinal cellular composition and transcriptomes. (A) UMAP clustering of retinal cell types across TS (*n* = 15), humans (*n* = 7), macaques (*n* = 4), mice (*n* = 7), and chicks (*n* = 8) retina. (B) Proportions of retinal cells in TS, humans, macaques, mice, and chicks. (C) Dendrogram based on the transcriptomic similarity of cell types across each species illustrates the conserved retinal cell types across species. (D) Dendrogram based on the transcriptomic similarity of retinal cell subtypes across species highlights the conserved subclusters among TS, humans, and macaques. ACs, amacrine cells; BCs, bipolar cells.

**Fig. 7.**
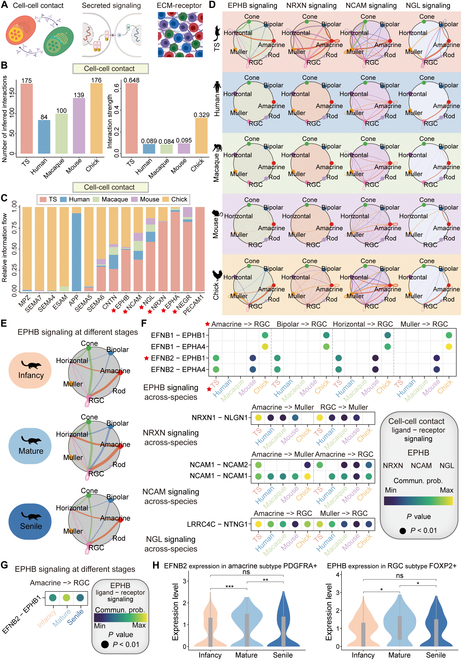
Cell communications across species. (A) Schematic graph of 3 cell communication methods including cell–cell contact, secreted signaling, and ECM–receptor. (B) Bar charts present the cross-species differences in the number and intensity of cell–cell contact in the retina. (C) Relative information flow of the retinal cells through the cell–cell contact way across different species. The red 5-pointed stars represent important signaling in the TS retina cells. (D) Networks visualize the intercellular communication of retinal cells in *EPHB*, *NRXN*, *NCAM*, and *NGL* signaling across humans, macaques, mice, and TS retinas. (E) *EPHB* signaling in TS retinal cells at different age stages. (F) Intensities of ligand–receptor pairs in the indicated cell types among different species. The red 5-pointed stars represent important ligand–receptor pair, cell communication, and TS. (G) Strength of *EFNB2*–*EPHB1* in *EPHB* signaling between ACs and RGCs of TS retina at different stages. (H) Expression of *EFNB2* in *PDGFRA*^+^ ACs and *EPHB* in *FOXP2*^+^ RGCs at different age stages. *P ≤ 0.05, **P ≤ 0.01, and ***P ≤ 0.001.

To study the differences and similarities between the TS retinas and those of other species, we integrated and analyzed the single-cell transcriptome profiles of 7 humans, 4 macaques, 7 mice, 8 chicks, and 15 TS from our study after strict quality control and homologous gene transformation (Materials and Methods). According to the UMAP analysis, the cell populations were well matched across multiple species, including rods, cones, ACs, BCs, RGCs, HCs, and Müller (Fig. 6A). While the overall cell types were conserved, the proportions of each cell type varied by species, where humans and macaques comprise proportionally more RGCs (Fig. 6B). The cross-species transcriptomic correlation analysis among the 5 species demonstrated a high degree of similarity across major retinal classes (Müller, rods, cones, HCs, BCs, RGCs, and ACs), where rods and cones of a given species are more closely related to those of other species than they are to other classes from the same species (Fig. S6A and Table S8). These findings are consistent with a recent single-cell study reporting that retinal cell classes and subclasses across 17 species (TS included) are highly conserved at the molecular level [44]. Additionally, relationships among species vary a lot by cell type. Humans and macaques are each other’s closest relatives in all these 6 cell types, particularly cones, which showed a large transcriptional similarity among TS, humans, and macaques (Fig. 6C and Fig. S6A). In our further analysis on cone subsets, we identified key DEGs such as PDE6H and CST3 that were consistently shared between TS and humans across both L-cone and S-cone subtypes, especially during the transition from mature to senile stages (Fig. S6B and C). These genes are involved in visual signal transduction and retinal protection against aging-related degeneration [45,46]. Notably, the shared differential expression of these genes highlights the potential of TS as a valuable model for studying cone-related retinal aging and degeneration. Meanwhile, RGCs, HCs, ACs, and BCs are more transcriptomically conserved across species, with RGCs exhibiting the least transcriptional divergence (Fig. S6A). Interestingly, the more remarkable transcriptional similarity of RGCs to ACs than to BCs among species was unveiled in our work, indicated by their clustering in the same subsidiary branch and increasing clumpiness index with age (Fig. S7A and B). Additionally, TS and chick retinal cells demonstrated greater transcriptomic similarity to each other than to those of humans or primates [13] (Fig. 7B).

When analyzing cell subtypes across species, we reclustered 37 subclasses of retinal cells and identified shared subclusters across TS, humans, and macaques, including C2, C3, C4, C6, C8, C11, C13, C21, C23, C25, C27, C28, C29, and C33. These crucial classes comprised key AC subtypes including *EPHA6*^+^, *PTPRT*^+^, *VIP*^+^, *DRD2*^+^, *PDGFRA*^+^, and *SSTR2*^+^, and BC subpopulations including *RELN*^+^, *FEZF2*^+^, *DSCAM*^+^, *PTPRQ*^+^, *TTR*^+^, and *ERBB4*^+^, which showed strong transcriptomic similarity to those in humans and macaques. In particular, *DRD2*^+^ ACs, *PTPRT*^+^ ACs, *PTPRQ*^+^ BCs, *ERBB4*^+^ BCs, and *TTR*^+^ BCs in the TS displayed greater similarity to humans than to macaques (Fig. 6D). We further compared species-specific genetic differences of TS retinal cells with the other 4 species, and the top 5 specific DEGs in each cell type were illustrated (Fig. S8A and B), indicating the least genetic specificity of TS RGCs across different species.

### TS-specific cell-to-cell communication among different retinal cell types

The cell bodies of retinal cells are organized into ONL, INL, and RGC layers, separated by 2 plexiform (synaptic) layers. Information flows through these layers in a defined direction: Photoreceptor cells in the ONL layer detect light and transmit visually evoked signals to interneurons in the INL layer. The interneurons (HCs, BCs, and ACs) process the information and provide it to retinal RGCs in the RGC layer. The RGCs then send axons through the optic nerve to the cerebral visual centers. Cellular communication is an essential process for the development and maintenance of all tissues, including the retina and nervous system. In general, cells employ 3 ways of communication: by secreting proteins or small-molecule compounds in the extracellular fluid (e.g., hormones, cytokines, and extracellular vesicles), by a special protein channel embedded in 2 adjacent cells, also known as intercellular channels, or through the direct contact of membrane surface molecules (e.g., binding of T cells and B cells) [47] (Fig. 7A).

As the results showed, distinct cell communications of retinal cells were exhibited among diverse species, especially interactions dominated by RGCs with ACs, BCs, and HCs (Fig. S9A). In the TS retina, most inferred interactions and the strongest interaction signal occur primarily through cell–cell contact, compared to the other 2 modes of cell communication (Fig. 7B and Fig. S9B and C). This aligns with the well-established understanding that retinal information transmission predominantly relies on synaptic neurotransmitter release [48,49]. With high interaction network weights, TSs were superior to other species in *EPHB*, *NCAM* (*NCAM1*-*NCAM1/2*), *NGL* (*LRRC4C*-*NTNG1*), *NRXN* (*NRXN*-*NLGN1*), *EPHA*, and *NEGR* signaling (*NEGR1*-*NEGR1*) via the cell–cell contact way (Fig. 7C and D), and *LAMININ* signaling (*LAMA4*-*SV2B*) through the ECM–receptor way, as well as *IGF* signaling (*IGF1*-*IGF1R*) but weak *NT* signaling (*BDNF*-*NTRK2*) through the secreted signaling way (Fig. S9D to G). In contrast, there is a lack of TS-specific *EPHB* signaling in humans and macaques, which has been previously revealed to increase the apoptosis of RGCs [50] (Fig. 7D). In addition, RGCs of TS established ligand–receptor associations with ACs, BCs, and HCs via *EPHB* signaling, wherein *EFNB2*–*EPHB1* and *EFNB2*–*EPHA4* pairs showed strong intensities between RGCs and ACs, RGCs and BCs, and RGCs and HCs in the TS, and the intensities weakened in the senile TS (Fig. 7E to G), along with decreased expression of *EFNB2* in *PDGFRA*^+^ ACs and *EPHB* in *FOXP2*^+^ RGC subtypes (Fig. 7H). Whether these cellular communications are related to proportional changes in these cell types in TS retina with age remains to be further elucidated; however, it provides us with potential hypotheses for studying the communication connections among retinal cells.

Finally, we examined retinal thickness in TS at different ages and found no apparent alteration in TS retinal thickness as age increased (Fig. S10A and B), strengthening the findings that specific cellular composition and communication of the TS retina contributes to resistance to age-related retinal degradation.

## Discussion

The ocular anatomy of the TS is partly similar to that of primates and humans. Hence, many studies have reported good application prospects of TS as a model for studying human retinal diseases such as glaucoma, axial myopia, and diabetic retinopathy [3,51–53]. In this study, a reference single-cell transcriptome atlas was generated, which revealed TS retinal cell type-specific characteristics during development. In complex retinal tissues, major cell types including cones, rods, BCs, ACs, HCs, RGCs, Müller, astrocytes, and microglia were classified, and the changes of cellular composition, novel cellular marker identification, and complex cell–cell communications in TS retina were also characterized with advancing age by cross-species analysis.

In our study, TS was shown to be a new species with an age effect on the cell subset composition and the gene expression pattern, which may provide support for the similar high-acuity vision and daily routine to human retina, also facilitating increased visual processing and excellent visual sharpness in the elderly TS [54–57]. Some special subpopulations of these cell types and marker genes were identified. We found that the cones comprised >90% of photoreceptor cells in the TS retina [58]. Humans and macaques possess trichromatic vision, characterized by 3 different types of cones expressing 3 distinct opsin proteins (S, M, and L) [23]. However, TSs are dichromatic, wherein their visual system includes 2 types of cone cells (S-cones and L-cones) that express *OPN1SW* and *OPN1LW*, respectively. Our immunostaining results revealed the expression of these 2 cellular markers of the cones in the ONL and RCL of the TS retina. In addition, the disease-related risk scoring showed a high correlation between the L-cones with color blindness and cone dystrophy. In contrast, a decline in rod proportions consistent with the decreased *CLUL1* expression with age in the TS retina was observed. *CLUL1* plays a physiological defense function to maintain cell viability and can promote rod survival after cellular injury by activating Akt and *STAT3*, but inactivating the pro-cellular death signal Bax [59,60]. Therefore, decreased expression of *CLUL1* may be associated with the reduction in rods in senile TS, which might represent another cellular marker of rods, also verified by our immunofluorescent staining. Moreover, high disease risk scores of rods with visual disorders imply that TS has great potential to mimic rod-related diseases such as RP, CSNB, and LCA; furthermore, the mechanistic role of *CLUL1* in rod-related deficits requires further investigation.

Different subtypes of BCs exhibit different functions. BCs, acting as interneurons, receive synaptic input from photoreceptors and transmit processed signals to RGCs via synaptic neurotransmitter release [12]. Quesada et al. [61] identified 14 BC types in the chicken retina based on dendritic morphology without considering axonal morphology. Another study confirmed the existence of at least 15 BC subtypes in the mouse retina using single-cell sequencing and experiments [61,62]. In mammals, BCs are usually divided into 2 types according to whether they respond to light depolarization (ON) or hyperpolarization (OFF): ON-BCs are dominated by rods, and OFF-BCs are dominated by cone cells [25]. In this study, we identified *CA10* as a novel marker for BCs in TS through triple immunofluorescent staining, revealing distinct expression patterns in both OFF_BCs (*CA10*^+^/*NETO1*^+^/*RBPMS*^−^) and ON_BCs (*CA10*^+^/*ISL1*^+^/*RBPMS*^−^) across different age stages. Identification of *CA10* enhances our understanding of BC subtype classification and their functional roles in retinal development and aging. Immunostaining results proved that OFF-BCs and ON-BCs were located in the INL layer of the TS retinas. As our results revealed, different from other OFF-BCs, the proportion of the *ERBB4*^+^ BC subpopulation exhibited an increased trend with age. The GO biological process analysis showed that this subpopulation mainly participated in regulating axon regeneration and the forming of inhibitory postsynaptic potentials. *ERBB4*, as a member of the *EGFR* subfamily of receptor tyrosine kinases, could promote inhibitory synapse formation, and the enhancing effects of *ERBB4*^+^ and *NRG* are vital for nervous system development [63]. In addition, PPI analysis also revealed the central role of regulation between *GRIA4* and *ERBB4*, indicating the critical roles of *ERBB4* in axon regulation [26,63–65].

In mammals, ACs are the most heterogeneous retinal cell type, which forms inhibitory synapses with BCs, RGCs, and other ACs [66]. From the perspective of neurochemical transmitters, ACs can be divided into 2 major categories: γ-aminobutyric acid (GABAergic) and glycine. GABAergic ACs generally have wide dendritic fields that laterally inhibit across larger areas of the retina, while glycinergic ACs have smaller, vertically oriented dendritic domains that mediate localized interactions between different layers of the inner plexiform lamina [67]. Our results validated 3 AC subtypes in the INL and RGC layers of TS retina: glycinergic (*SLC6A9*^+^), SACs (*CHAT*^+^), and *TAC1*^+^ ACs. SACs are the only cholinergic neurons in the retina, and cholinergic activity plays an important role in the formation of deep retinal vascular plexuses and maintains the stability of the retinal vascular structure together with Müller glia, astrocytes, endothelial cells, and pericytes [68]. Also, as GO analysis indicated, SACs are primarily involved in acetylcholine biosynthesis. Cell-to-cell communication showed that SACs exhibited the most cell interactions with other cell types at the mature stage of TS. In addition, the expression of cholinergic neuron markers (*CHAT* and *MEGF11*) in SACs showed the same trend as the cellular communication of SACs with corresponding alternated ligand signaling, such as *VEGF*, *FGF*, and *PDGF* (Fig. 4H and I), further indicating the critical role of SACs in the source of acetylcholine in the retina and might jointly regulate retinal development and angiogenesis in blood vessel-associated cells in retinal disorders [62,69].

More than 30 types of RGCs have been identified in previous studies [34], but the exact role in image formation remains to be determined in TS. We identified 4 TS-specific RGC subpopulations, including *SLIT2*^+^, *RUNX1*^+^, *FOXP2*^+^, and *SPP1*^+^ cells, all located in the RGC layer of the TS retina, suggestive of TS-specific RGC markers. *FOXP* can define different types of neurons in the brain and spinal cord. It also plays an essential role in regulating the plasticity of neural circuits [70,71]. Rousso et. al. previously proposed a subtype of RGCs that could express *FOXP2*, which is different from known RGC subtypes, including α-RGCs, ip-RGCs, J-RGCs, and ooDSGCs [72]. *SLIT2* is an RGC growth cone collapse factor that inhibits RGC axon guidance. Importantly, *ROBO1* and *SLIT2* jointly promote RGC axon growth and play roles in neuronal axon guidance, angiogenesis, inflammatory cell chemotaxis, and tumor cell migration and transfer. Generally, RGCs project axons to their brain targets. The axons of the left and right eyes then meet in the optic chiasm and cross most of the contralateral side. Damage to RGC or axons results in impaired energy metabolism, which leads to optic nerve lesions (e.g., glaucoma) and even blindness [73]. The survival rate varies significantly among different RGC subtypes, wherein α-RGC has the highest survival rate and axon regeneration rate [74]. *SPP1*, known as osteopontin (*OPN*), a marker for α-RGCs, is a secreted glycosylated phosphoprotein that can affect cell survival, inflammation, migration, and homeostasis after injury [36]. *SPP1* was first morphologically identified in the cat retina and belongs to a subset of intrinsically photosensitive RGCs (ipRGCs) [75]. The loss of *SPP1* during development can lead to a decrease in the number of RGCs [36]. Therefore, *SPP1* may be an important target in RGCs for treating retinal degenerative diseases. Although we were unable to characterize the ipRGC marker *OPN4* in TS, C9 was considered an M4-type ipRGC because of the co-expression of interleukin-1 receptor accessory protein-like 2 (*IL1RAPL2*) [76]. We also found a higher glaucoma disease score in α-RGC (C8, C9) than in other RGC subpopulations, suggesting the potentials of TS in investigating the pathogenesis of glaucoma involving C8 and C9 RGC subclasses.

HCs, specifically H1 and H2 HCs, are specialized neurons found in the retina of vertebrate eyes [77]. H1 receives input from photoreceptors (rods and cones) and provides lateral inhibition to neighboring photoreceptors, enhancing contrast and sharpening the edges of visual stimuli [38]. H2, receiving input from cones [38], has a primarily inhibitory function and modulates the communication between photoreceptors and BCs, which provide feedback inhibition to photoreceptors, regulating the sensitivity and dynamic range of the retinal circuitry [78]. As shown in our results, the proportion of H1 exhibited a decreased and H2 an increased trend from infant to senile periods. GO analysis revealed that H1 is closely related to dendrite morphogenesis, regulation of guanosine triphosphatase activity, dendritic spine development, peptidyl-serine dephosphorylation, and myelination in the peripheral nervous system, while H2 in the senile group is closely related to axon development, axogenesis, regulation of cell development, neural retina development, and developmental cell growth. Thus, these biological processes may be associated with the visual processing and perception of H1 and H2 HCs [12,40,42]. Additionally, our findings reveal the presence of 2 distinct types of HCs in TS, a pattern that mirrors what has been observed in primates. These 2 HC subtypes demonstrated strong homology to those in primates, particularly in terms of their gene expression profiles and functional properties. This close homology suggested that TS can serve as an effective model for studying retinal circuits and cellular organization that are highly relevant to primate and human vision. The identification of these 2 HC subtypes in TS further underscores the evolutionary conservation of retinal cell types across species. It highlights the translational potential of using TS in retinal research, especially for understanding how these cells contribute to visual processing and how similar mechanisms might apply in humans.

To study the differences and similarities between the TS retina and that of other species, we integrated and analyzed our TS single-cell transcriptome profiles with humans, macaques, mice, and chicks. A recent study integrating single-cell transcriptomic cell atlases of the retina from 17 vertebrate species (TS included) reported that the retinal cell classes and subclasses are highly conserved at the molecular level through evolution, mirroring their structural and functional conservation [44]. Our study consistently revealed the conservation of retinal cell classes across different species. However, we found that the proportions of each cell type in the different species varied, especially for that of TS ACs, BCs, and cones, which accounted for the significant proportions; ACs, BCs, RGCs, and Müller compose the most cell types in macaques and humans; in contrast, BCs, rods, and ACs are the main cell types in mice, and BCs and ACs are the main cell types in chicks. Based on species analysis, relationships among species also vary by cell clusters. Humans and macaques are each other’s closest relatives in most of the main cell types in the retina, while retinal cells from TS and chick showed more transcriptomical similarity in overall major cell populations to each other than either to humans or primates. This outcome might be explained by the more complex cellular composition of cell subtypes in TS and chick than humans or macaques retina [13]. Nevertheless, the cones of TS exhibited a large transcriptional similarity to humans and macaques, indicating that TS can be used in cone-related research like color blindness and cone dystrophy [79] (Fig. 2H). Notably, the shared differential expression of genes such as PDE6H and CST3 in L-cone and S-cone subtypes between humans and TS in the mature and senile stages highlights the potential of TS as a valuable model for studying cone-related retinal aging and degeneration, offering insights into human retinal diseases. Additionally, great similarities were uncovered in these TS retina subtypes including *DRD2*^+^ ACs, *PTPRT*^+^ ACs, *PTPRQ*^+^ BCs, *ERBB4*^+^ BCs, and *TTR*^+^ BCs to those of humans and macaques. Therefore, these similarities in retinal classes and subtypes support the idea that the future retinal investigations could focus on the cones and subtypes of ACs and BCs to facilitate the conceptual advance in retinal research field. We also explored key molecules with large species-specific differences between TS retinas and other species. The top 5 specific DEGs in each cell type reflected the specificity of the TS retina in each cell population, especially for the Müller and RGCs. These type-specific transcriptional programs can provide the basis for later research on TS.

Cell-to-cell communication in the retina is vital in maintaining normal tissue development and balancing retinal structure and function. CellChat was used to analyze the communication among all cell types. Based on the multimer ligand–receptor complex, CellChat predicts the differential expression of cell–cell interactome gene pairs by evaluating gene expression across cell types and quantitatively infers and analyzes cell-to-cell communication [80]. In this study, we integrated snRNA-seq datasets from humans, macaques, mice, chicks, and TS to better understand interspecies differences in cell–cell interactions. RGCs represent an important communication channel between the retina and the brain and are the only projection neurons of the retina. RGCs are the dominant communicating cells in all species and are involved in 3 communication ways: cell–cell contact, secreted signaling, and ECM–receptor. Our results showed that intercellular communication presented a similarity to the transcriptional characteristics, and more remarkable similarities existing among RGCs, ACs, BCs, HCs, and RGCs exhibited higher interaction weights with ACs, BCs, and HCs through the cell–cell contact way in the TS than humans, macaques, and mice. The intercellular communication was also presented between RGCs and cones, which may be explained by the fact that CellChat also performs in distinguishing spatially distant cells in terms of both the number of interactions and the interaction strengths [80].

Most importantly, we found that 2 ligand–receptor pairs *EFNB2*–*EPHB1* and *EFNB2*–*EPHA4* in *EPHB* signaling, which can promote RGC apoptosis [81], are strengthened in the interactions of RGCs communicating with BCs, ACs, and HCs, but little in humans and macaques. These may be one of the reasons why RGCs comprise the major cell types in the retina of macaques and humans [15], which needs further investigation. The weakening interaction intensity of *EPHB* signaling in the senile TS might constitute the proportional increase of *FOXP2*^+^ RGCs in the aged TS retina. Studies to test these possibilities are underway. Overall, our cross-species outcomes concluded that retinal cellular and subcellular composition are highly conserved at the molecular level through evolution, and several lines of evidence, such as distinct cellular communication patterns and special ligand–receptor interaction signaling, support the cellular specificity in the TS retina.

### Conclusions

Our study produced a comprehensive snRNA-seq atlas for deciphering the cellular and genetic landscapes of the TS retina at the infant, mature, and senile stages. Changes in cellular proportions and cell type-specific molecular alterations during development and aging are well understood in the TS retina. While previous studies have primarily focused on single aspects of retinal development or gene expression in TS, our work represents the first to comprehensively investigate the histology and gene expression profiles of the TS retina across the postnatal lifespan. Importantly, our work highlights the high degree of conservation in retinal cell types and gene expression patterns among TS, humans, and macaques, offering valuable insights into the evolutionary aspects of retinal function. Particularly, we observed from cross-species analysis that TS cones are more similar to that of humans and macaques than to other species, suggesting TS as a more relevant model for studying cone-dominant diseases, particularly given that mice photoreceptors are primarily rods. Moreover, the consistently shared DEGs between TS and humans across both L-cone and S-cone subtypes with aging underscore the potential of TS as a valuable model for studying age-related retinal disorders, particularly those involving cone degeneration. Furthermore, our study identified unique, TS-specific cell–cell communications pathways between RGCs and interneurons, which may explain the species-specific differences in retinal cellular composition. These findings provide critical insights into the molecular and cellular mechanisms underlying age-related changes in the TS retina and offer a bridge between nonhuman primate and rodent models in the study of retinal deficits. The cellular and molecular insights gained from this study offer reliable theoretical support for future research on retinal diseases, contributing to the development of preventative and therapeutic strategies.

## Materials and Methods

### Ethical statement

All experimental procedures were approved by the Animal Care and Use Committee of Kunming Medical University (approval no. 20221868). This study was performed in accordance with the Principles for the Ethical Treatment of TS.

### TS retina tissue collection

Infancy and mature TSs were provided by the Animal Centre of Kunming Medical University and housed in individual cages under a 12-h light/dark cycle, with food and water available throughout the study. Senile TSs were purchased from the Institute of Zoology (Chinese Academy of Sciences). Age and related information of TS used in this study was provided in Fig. S1A and Table S9. Retinal tissues were carefully dissected from the TS eyeballs removed from infancy, mature, and senile animals. Tissues used for histological analysis were fixed in 4% paraformaldehyde, and those used for snRNA-seq were frozen in liquid nitrogen.

### Nuclei isolation and snRNA-seq on the 10× Genomics platform

The frozen tissues were first thawed, cut into small pieces, and homogenized using a glass Dounce tissue grinder (catalog no. D8938, Sigma). The tissue was then homogenized 25 times with pestle A and 25 times with pestle B in 2 ml of ice-cold nuclei EZ lysis buffer. The sample was therefore incubated on ice for 5 min, with an additional 3 ml of cold EZ lysis buffer. Subsequently, nuclei were centrifuged at 500*g* for 5 min at 4 °C, washed with 5 ml of ice-cold EZ lysis buffer, and incubated on ice for 5 min. After centrifugation, the nucleus pellet was washed with 5 ml of nuclei suspension buffer [NSB; consisting of 1× phosphate-buffered saline (PBS), 0.01% bovine serum albumin (BSA), and 0.1% ribonuclease inhibitor (catalog no. 2313A, Clontech)]. Afterward, isolated nuclei were resuspended in 2 ml of NSB, filtered through a 35-μm cell strainer (catalog no. 352235, Corning-Falcon), and counted. Finally, a concentration of 1,000 nuclei per microliter was employed for loading on a 10× channel.

### Construction of snRNA-seq library and processing and quality control of snRNA-seq data

We prepared snRNA-seq libraries with Chromium Next GEM Single Cell 3′ Reagent Kits v3.1 on the Chromium Controller (10× Genomics). The single nucleus was suspended in PBS containing 0.04% BSA. The nuclei suspension was then loaded onto the Chromium Next GEM Chip G, and the Chromium Controller was run to generate single-cell gel beads in the emulsion (GEMs) according to the manufacturer’s recommendation. Captured nuclei were lysed, and the released RNA was barcoded through reverse transcription in individual GEMs. Barcoded, full-length cDNA was generated, and libraries were constructed according to the performer’s protocol. The quality of libraries was assessed by Qubit 4.0 and Agilent 2100. Sequencing was performed on Illumina NovaSeq 6000 with a sequencing depth of at least 50,000 reads per nucleus and 150-base pair (PE150) paired-end reads (performed by Biomarker Technologies Corporation, Beijing, China). Then, we performed alignment to this amended reference using 10× Cell Ranger v7.0, which employs the STAR sequence aligner. The reference genome was the TS_3.0 genome (http://www.treeshrewdb.org/download.html). We determined gene expression counts using unique molecular identifiers (UMIs) for each cell barcode–gene combination. Following alignment, we filtered cell barcodes to identify those that contain nuclei using the approach implemented in Cell Ranger v7.0, and only these barcodes were considered for downstream analysis including clustering and cell-type identification as well as differential expression analysis by Seurat (v4.2.0). To remove the nuclei with low quality, nuclei with gene number over 200 but less than 6000 and the ratio of mitochondria lower than 5% were maintained, and genes with at least one feature count in more than 3 nuclei were used for the following analysis. Doublets were detected using DoubletFinder (version 2.0.3) [82], referring to the official recommendation of Doublet Rate < number of cells in a single sample * 8 * 1e−6 (calculated by increasing the double cell ratio by 8‰ for every 1000 cells added). After removing doublets, we filtered cells expressing multiple cell markers and nonretinal cells from the integrated data. In addition, we further removed meaningless double cells during cell subtype analysis. After sample integration and clustering, clusters lacking specific marker genes, relatively low gene content, and high mitochondrial ratios were discarded.

### Integration, clustering, and identification of cell types

After quality control, a typical workflow for integrating multiple sample data using the LIGER software package consisted of the following steps: First, each sample datum was normalized to account for differences in total gene-level counts across cells using the “NormalizeData” function. Next, “ScaleData” function was used to normalize datasets without centering based on the mean, thereby obtaining nonnegative input data. Next, we performed integrative nonnegative matrix factorization (iNMF) on the normalized and scaled snRNA-seq data to identify shared and dataset-specific factors. To run iNMF on the scaled datasets, we used the “optimizeALS” function with proper hyperparameter settings (*k* = 20). We then performed quantile normalization by dataset, factor, and cluster to fully integrate the datasets (using “QuantileNorm” function, knn *k* = 20, quantiles = 50). After data integration and scaling, principal components analysis (PCA) was applied with the “RunPCA” function, and appropriate PCs were selected for the following analysis. Dimensionality was reduced with the “RunUMAP” function. “FindNeighbors” and “FindClusters” [83] functions were applied to perform clustering. Marker genes for each cluster were identified by the “FindAllMarkers” function with the cutoff of adjusted *P* < 0.05 and |log_2_FC| > 0.25. Canonical marker genes were used to identify the cell types.

### Identification of DEGs across clusters

In our dataset, 107,718 single cells were filtered and grouped into 29 clusters. The clusters were annotated using known marker genes (Table S1). We identified cones, rods, BCs, ACs, HCs, microglia, Müller glia, and astrocytes. DEGs were identified using “FindMarkers” function implemented in Seurat v4 [84] across clusters with the options “logfc.threshold = 0.25, min.pct = 0.1”. *P* values were corrected using the Bonferroni method, and 0.05 was set as a threshold to define significance.

### Quantification of the number for cell types in infancy, mature, and senile samples

To get insight into the changes of cell types during retinal aging of TS, we performed the cell number analysis following the method and formula reported by Schirmer et al. [63,85]. In detail, the number of nuclei in each cluster and individual was normalized to the total number of nuclei captured from each individual. Then, a one-way analysis of variance (ANOVA) was used to make comparisons of normalized cell numbers and cell types for individuals at different age stages.

### Differential gene expression analysis using linear mixed-model regression

To identify genes differentially expressed among infancy, mature, and senile samples per cell type, *P* values were calculated and false discovery rate (FDR) corrected using model-based analysis of single-cell transcriptomics (MAST). All nuclei from infancy, mature, and senile samples for corresponding cell types were used. MAST was used to perform zero-inflated regression analysis by fitting a linear mixed model. To exclude gene expression changes stemming from confounders, such as age, sex, and fractions of ribosomal and mitochondrial transcripts, the following model was fit with MAST:

zlm( ∼ group + age + sex + riboperc + mitoperc, sca, method = glmer, ebayes = F, silent = T)

To identify genes differentially expressed due to the age effect, the likelihood ratio test was performed by comparing the model with and without the diagnosis factor. Genes with at least 10% increase or decrease in expression and an FDR-corrected *P* < 0.05 were selected as differentially expressed.

### GO enrichment analysis

The “enrichGO” function of clusterProfiler R package [86] was used for enrichment analysis, and the Benjamini-and-Hochberg (BH) method was employed for multiple test correction (OrgDb = org.Hs.eg.db, pAdjustMethod = “BH”, pvalueCutoff = 0.05). A GO term with an adjusted *P* value of lower than 0.05 was considered significantly enriched. Of note, “org.Hs.eg.db” package was used due to the homogeneity between TS and humans and the lack of comprehensive TS resources.

### Integration, clustering, and identification of cell types across species

We compared the previously reported mice [16], chicks [13], macaques [18], and humans [19] retinal snRNA-seq datasets, with the TS retinal snRNA-seq dataset generated in this study (Table S10). First, we assembled cells from our dataset with those from published single-cell RNA-sequencing datasets of other 4 species based on orthologous genes identified among the 5 species genomes with the R package homologene. The homologous genes are 1–1 matched in 5 species, excluding genes with ambiguity and those that did not have corresponding orthologs (Table S11). Then, a cross-species comparison was performed. Seurat object of each species sample was constructed from the decontaminated matrix, and unsupervised clustering was performed to identify cellular subpopulations (https://satijalab.org/seurat/) [87]. Datasets from different sequencing libraries were subjected to normalization [using “NormalizeData()” function with parameters “normalization.method = “LogNormalize”, reduction = “rpca”] and identification of highly variable genes (HVGs) (using “FindVariableFeatures” function with the options “selection.method = “vst”, nfeatures = 3000”). Then, we applied “FindIntegrationAnchors” and “IntegrateData” functions to integrate all sequencing libraries with the top 50 significant PCs (dim = 1:50). The top 3,000 HVGs of each dataset were used for the downstream PCA. The top 20 significant PCs were selected for clustering and visualization using a UMAP. Cell identities were annotated based on the expression of canonical cell-type markers. When data from different species are integrated, dendrograms for the cell-averaged profiles were constructed using hclust (R package “stats”) and then plotted in a circular representation using the circlize_dendrogram function (R package “dendextend”).

### Visualization of cell echelon status using TooManyCells

To visualize the relationships in the evolutionary branches of cells and significant differences among the 5 species, we performed a clustering analysis of retinal snRNA-seq data using TooManyCells (v2.2.0.0, Linux) [88]. For visualization purposes, the tree projection was trimmed by a median absolute deviation derived from the median size of all nodes of the original tree using the TooManyCells smart cutoff parameter. In particular, we set the minimum size of each leaf to 10 cells and colored each leaf according to the cell type.

### Construction of cellular communication network

Intercellular communication was analyzed using the CellChat (v1.4.1) R package with default parameters. TS retina datasets for infancy, mature, and senile animals and humans, macaques, mice, and chicks retinal datasets were analyzed separately, and intercellular communication analysis was performed based on cell types. Cell–cell communication network was visualized using the “netVisual_aggregate” function, the centrality score was computed and visualized using the “netAnalysis_signalingRole_network” function, and the relative contribution of each ligand–receptor pair was visualized using the “netAnalysis_contribution” function.

### RNA velocity analysis

We first applied velocyto [89] to count the abundances of unspliced and spliced transcripts using the bam output of CellRanger. We then applied scVelo [90] to find variable genes, calculated RNA velocities via dynamical models, and visualized the velocities on the UMAP embeddings where all samples were integrated together using the Seurat pipelines described above.

### Single-cell regulatory network inference and clustering

To perform transcription factor network inference, the data were subsampled by randomly selecting cells from each cell type or cellular subpopulation. Analyses were performed using the single-cell regulatory network inference and clustering (SCENIC) R package (version 1.1.0, which corresponds to RcisTarget 1.2.0 and AUCell 1.4.1). The activity of the regulatory networks was evaluated using the full dataset during the scoring step using AUCell (step 3). Regulons annotated as “extended” included target genes harboring motifs that had been linked to the respective transcription factor by lower confidence annotations [91].

### Gene set score analysis

Gene sets related to “Retinitis pigmentosa”, “Congenital stationary night blindness”, “Color blindness”, “Leber’s congenital amaurosis”, “Cone dystrophy”, and “Glaucoma” were obtained from the DisGeNET database (https://www.disgenet.org/home/). Gene set scores were acquired by analyzing the transcriptome of each input cell against the aforementioned gene sets using the Seurat function “AddModuleScore.” Changes in the scores between infancy, mature, and senile samples were analyzed using the ggpubr R package via the Wilcoxon test.

### Mfuzz analysis

Mfuzz analysis was used to detect the change trend of cell-specific genes during retina aging. First, nuclei corresponding to the given cell type were selected from the full dataset. Second, raw counts were summed in order to produce a “pseudo-bulk” transcriptome for each group. Third, through the cluster analysis of expression patterns, the gene expression trend of infancy, mature, and senile TS retina samples was presented.

### Optical coherence tomography

TSs in the infant, mature, and senile groups were anesthetized using an intraperitoneal injection of 3% phenobarbital (50 mg/kg). Compound tropicamide eye drops were applied externally to dilate the pupil, and flufloxacin was used to protect the cornea during and after surgery. TSs were photographed using a Micron IV retinal imaging camera (Phoenix Research Laboratory, Pleasanton, CA, USA). Finally, the retinal thicknesses of the optical coherence tomography (OCT) images were measured using the ImageJ software.

### Multiplexed immunofluorescence staining

Retinal tissues from 3 infancy, 3 mature, and 3 senile TS were used for multiplex immunofluorescence staining. The retinal tissues were dissected and fixed with 4% paraformaldehyde (catalog no. BL539A, Biosharp) for up to 24 h and sequentially placed in 10%, 20%, and 30% sucrose (catalog no. 21164955, Biosharp) at 4 °C until the tissues were completely immersed in each solution. The tissue samples were frozen with optimal cutting temperature compound (catalog no. 4583, Tissue-Tek) at −40 °C for 30 min and sectioned at a thickness of 15 μm using a microtome (catalog no. CM1950, Leica). Sections were then stored at −40 °C. After antigen repair with citrate-EDTA antigen retrieval solution (1×) (catalog no. BL55A, Biosharp), the sections were incubated with 3% hydrogen peroxide (Jiangxi Grass Coral Disinfection Products Co. Ltd.) for 15 min and blocked with 5% goat serum (catalog no. SL038, Solarbio) and 0.3% Triton X-100 (catalog no. 1139ML100, BioFROX) for 2 h. Subsequently, the sections were supplemented with primary antibodies for 18 h at 4 °C. This was followed by rinsing in PBS with Tween 20 (PBST) 5 times (5 min each). Secondary antibodies (catalog no. kit-5020, MaxVision-HRP, mouse/rabbit) were incubated at room temperature for 15 min. This was followed by washing with PBST and incubation for 10 min with TSAPlus fluorescent enhancement dye (iF488-Tyramide, iF555-Tyramide, or iF647-Tyramide) (catalog no. GB1236, Servicebio). For double/triple-label staining, these sections were subjected to antigen repair again with citrate-EDTA antigen retrieval solution and were incubated with 3% hydrogen peroxide for 15 min, followed by blocking with 5% goat serum and 0.3% Triton X-100. Incubation with primary and secondary antibodies was performed as described previously. Finally, after counterstaining with 4′,6-diamidino-2-phenylindole (DAPI) for 10 min, sections were sealed with an anti-fluorescent quenching agent (catalog no. Po126, Beyotime). Using a confocal microscope (NIS-Elements AX) with 2,048 × 2,048 pixel, 3 to 5 sections of each group were randomly selected to observe the positive cells. Detailed primary antibody information is provided in Table S12.

### Quantification and statistical analysis

SPSS Statistics 26.0 (IBM, Chicago, IL, USA) was used to compare the data from TS at different age stages. Statistical analyses were performed using one-way ANOVA with corresponding post hoc tests for multiple group comparison following the normality and homogeneity tests. Graphical visualization was performed using GraphPad Prism software (v 9.1.1). The sample size and *P* values are given in the figure legends. *P* values were designated as follows: *P ≤ 0.05, **P ≤ 0.01, ***P ≤ 0.001, ****P ≤ 0.0001.

## Data Availability

The raw sequence data reported in this paper have been deposited in the Gene Expression Omnibus of the National Center for Biotechnology Information, under accession number GSE227524, and are accessible at https://www.ncbi.nlm.nih.gov/geo/query/acc.cgi?acc=GSE227524 . All data supporting the findings of this study are provided within the paper and its Supplementary Materials. Source data are provided with this paper.

## References

[1] Wang YY, Wang JD, Wang L, Dan QQ, Xia QJ, Wang TH, Xiong LL. Establishment of neurobehavioral assessment system in tree shrew SCT model. J Mol Neurosci. 2020;70(3):308–319. 31845102 10.1007/s12031-019-01414-9

[2] Lin N, Xiong LL, Zhang RP, Zheng H, Wang L, Qian ZY, Zhang P, Chen ZW, Gao FB, Wang TH. Injection of Aβ1-40 into hippocampus induced cognitive lesion associated with neuronal apoptosis and multiple gene expressions in the tree shrew. Apoptosis. 2016;21(5):621–640. 26897171 10.1007/s10495-016-1227-4

[3] Gorbatyuk OS, Pitale PM, Saltykova IV, Dorofeeva IB, Zhylkibayev AA, Athar M, Fuchs PA, Samuels BC, Gorbatyuk MS. A novel tree shrew model of diabetic retinopathy. Front Endocrinol. 2021;12: Article 799711. 10.3389/fendo.2021.799711 PMC8762304 35046899

[4] Tanabe S, Fu J, Cang J. Strong tuning for stereoscopic depth indicates orientation-specific recurrent circuitry in tree shrew V1. Curr Biol. 2022;32(24):5274–5284.e6. 36417902 10.1016/j.cub.2022.10.063 PMC9772061

[5] Fan Y, Luo R, Su LY, Xiang Q, Yu D, Xu L, Chen JQ, Bi R, Wu DD, Zheng P, et al. Does the genetic feature of the Chinese tree shrew (Tupaia belangeri chinensis) support its potential as a viable model for Alzheimer’s disease research? J Alzheimers Dis. 2018;61(3):1015–1028. 29332044 10.3233/JAD-170594

[6] Wang J, Azimi H, Zhao Y, Kaeser M, Vaca Sánchez P, Vazquez-Guardado A, Rogers JA, Harvey M, Rainer G. Optogenetic activation of visual thalamus generates artificial visual percepts. eLife. 2023;12:e90431. 37791662 10.7554/eLife.90431 PMC10593406

[7] Mustafar F, Harvey MA, Khani A, Arató J, Rainer G. Divergent solutions to visual problem solving across mammalian species. eNeuro. 2018;5(4):ENEURO.0167-18.2018. 10.1523/ENEURO.0167-18.2018 PMC6071193 30073190

[8] Li C, McHaney KM, Sederberg PB, Cang J. Tree shrews as an animal model for studying perceptual decision-making reveal a critical role of stimulus-independent processes in guiding behavior. eNeuro. 2022;9(6):ENEURO.0419-22.2022. 10.1523/ENEURO.0419-22.2022 PMC9718354 36414413

[9] Müller B, Peichl L, De Grip WJ, Gery I, Korf HW. Opsin- and S-antigen-like immunoreactions in photoreceptors of the tree shrew retina. Invest Ophthalmol Vis Sci. 1989;30(3):530–535. 2466810

[10] Brunke J, Russo IRM, Orozco-terWengel P, Zimmermann E, Bruford MW, Goossens B, Radespiel U. Dispersal and genetic structure in a tropical small mammal, the Bornean tree shrew (Tupaia longipes), in a fragmented landscape along the Kinabatangan River, Sabah Malaysia. BMC Genet. 2020;21(1):43. 32303177 10.1186/s12863-020-00849-z PMC7164274

[11] Schumacher JW, McCann MK, Maximov KJ, Fitzpatrick D. Selective enhancement of neural coding in V1 underlies fine-discrimination learning in tree shrew. Curr Biol. 2022;32(15):3245–3260.e5. 35767997 10.1016/j.cub.2022.06.009 PMC9378627

[12] Masland RH. The neuronal organization of the retina. Neuron. 2012;76(2):266–280. 23083731 10.1016/j.neuron.2012.10.002 PMC3714606

[13] Yamagata M, Yan W, Sanes JR. A cell atlas of the chick retina based on single-cell transcriptomics. eLife. 2021;10:e63907. 33393903 10.7554/eLife.63907 PMC7837701

[14] Mahato B, Kaya KD, Fan Y, Sumien N, Shetty RA, Zhang W, Davis D, Mock T, Batabyal S, Ni A, et al. Pharmacologic fibroblast reprogramming into photoreceptors restores vision. Nature. 2020;581(7806):83–88. 32376950 10.1038/s41586-020-2201-4 PMC7469946

[15] Baden T, Euler T, Berens P. Understanding the retinal basis of vision across species. Nat Rev Neurosci. 2020;21(1):5–20. 31780820 10.1038/s41583-019-0242-1

[16] Macosko EZ, Basu A, Satija R, Nemesh J, Shekhar K, Goldman M, Tirosh I, Bialas AR, Kamitaki N, Martersteck EM, et al. Highly parallel genome-wide expression profiling of individual cells using nanoliter droplets. Cell. 2015;161(5):1202–1214. 26000488 10.1016/j.cell.2015.05.002 PMC4481139

[17] Tran NM, Shekhar K, Whitney IE, Jacobi A, Benhar I, Hong G, Yan W, Adiconis X, Arnold MKE, Lee JM, et al. Single-cell profiles of retinal ganglion cells differing in resilience to injury reveal neuroprotective genes. Neuron. 2019;104(6):1039–1055.e12. 31784286 10.1016/j.neuron.2019.11.006 PMC6923571

[18] Peng YR, Shekhar K, Yan W, Herrmann D, Sappington A, Bryman GS, van Zyl T, do MTH, Regev A, Sanes JR. Molecular classification and comparative taxonomics of foveal and peripheral cells in primate retina. Cell. 2019;176(5):1222–1237.e22. 30712875 10.1016/j.cell.2019.01.004 PMC6424338

[19] Yan W, Peng YR, van Zyl T, Regev A, Shekhar K, Juric D, Sanes JR. Cell atlas of the human fovea and peripheral retina. Sci Rep. 2020;10(1):9802. 32555229 10.1038/s41598-020-66092-9 PMC7299956

[20] Lu C, Sun XM, Li N, Wang WG, Kuang DX, Tong PF, Han YY, Dai JJ. CircRNAs in the tree shrew (Tupaia belangeri) brain during postnatal development and aging. Aging. 2018;10(4):833–852. 29723158 10.18632/aging.101437 PMC5940110

[21] Kulesh B, Reese BE, Keeley PW. Contraction of axonal and dendritic fields in Sox5-deficient cone bipolar cells is accompanied by axonal sprouting and dendritic hyper-innervation of pedicles. Front Neuroanat. 2022;16: Article 944706. 36093292 10.3389/fnana.2022.944706 PMC9459848

[22] Wu F, Li R, Umino Y, Kaczynski TJ, Sapkota D, Li S, Xiang M, Fliesler SJ, Sherry DM, Gannon M, et al. Onecut1 is essential for horizontal cell genesis and retinal integrity. J Neurosci. 2013;33(32):13053–13065. 23926259 10.1523/JNEUROSCI.0116-13.2013 PMC3735885

[23] Muller B, Peichl L. Topography of cones and rods in the tree shrew retina. J Comp Neurol. 1989;282(4):581–594. 2723153 10.1002/cne.902820409

[24] Kim YK, Lee H, Ismail T, Kim Y, Lee HS. Dach1 regulates neural crest migration during embryonic development. Biochem Biophys Res Commun. 2020;527(4):896–901. 32430182 10.1016/j.bbrc.2020.05.009

[25] Euler T, Haverkamp S, Schubert T, Baden T. Retinal bipolar cells: Elementary building blocks of vision. Nat Rev Neurosci. 2014;15(8):507–519. 25158357 10.1038/nrn3783

[26] Paz JT, Bryant AS, Peng K, Fenno L, Yizhar O, Frankel WN, Deisseroth K, Huguenard JR. A new mode of corticothalamic transmission revealed in the Gria4(-/-) model of absence epilepsy. Nat Neurosci. 2011;14:1167–1173. 21857658 10.1038/nn.2896 PMC3308017

[27] Wang H, Liu F, Chen W, Sun X, Cui W, Dong Z, Zhao K, Zhang H, Li H, Xing G, et al. Genetic recovery of ErbB4 in adulthood partially restores brain functions in null mice. Proc Natl Acad Sci USA. 2018;115(51):13105–13110. 30498032 10.1073/pnas.1811287115 PMC6304932

[28] Rahman-Enyart A, Lai C, Prieto AL. Neuregulins 1, 2, and 3 promote early neurite outgrowth in ErbB4-expressing cortical GABAergic interneurons. Mol Neurobiol. 2020;57(8):3568–3588. 32542595 10.1007/s12035-020-01966-7

[29] Cheng CW, Chow RL, Lebel M, Sakuma R, Cheung HOL, Thanabalasingham V, Zhang X, Bruneau BG, Birch DG, Hui CC, et al. The Iroquois homeobox gene, Irx5, is required for retinal cone bipolar cell development. Dev Biol. 2005;287(1):48–60. 16182275 10.1016/j.ydbio.2005.08.029

[30] Erickson T, French CR, Waskiewicz AJ. Meis1 specifies positional information in the retina and tectum to organize the zebrafish visual system. Neural Dev. 2010;5:22. 20809932 10.1186/1749-8104-5-22 PMC2939508

[31] Sung MS, Heo H, Eom GH, Kim SY, Piao H, Guo Y, Park SW. HDAC2 regulates glial cell activation in ischemic mouse retina. Int J Mol Sci. 2019;20(20):5159. 31627491 10.3390/ijms20205159 PMC6829428

[32] Welsbie DS, Mitchell KL, Jaskula-Ranga V, Sluch VM, Yang Z, Kim J, Buehler E, Patel A, Martin SE, Zhang PW, et al. Enhanced functional genomic screening identifies novel mediators of dual leucine zipper kinase-dependent injury signaling in neurons. Neuron. 2017;94:1142–1154.e6. 28641113 10.1016/j.neuron.2017.06.008 PMC5553555

[33] Lam JD, Oh DJ, Wong LL, Amarnani D, Park-Windhol C, Sanchez AV, Cardona-Velez J, McGuone D, Stemmer-Rachamimov AO, Eliott D, et al. Identification of RUNX1 as a mediator of aberrant retinal angiogenesis. Diabetes. 2017;66(7):1950–1956. 28400392 10.2337/db16-1035 PMC5482092

[34] Rheaume BA, Jereen A, Bolisetty M, Sajid MS, Yang Y, Renna K, Sun L, Robson P, Trakhtenberg EF. Single cell transcriptome profiling of retinal ganglion cells identifies cellular subtypes. Nat Commun. 2018;9(1):2759. 30018341 10.1038/s41467-018-05134-3 PMC6050223

[35] Niclou SP, Jia L, Raper JA. Slit2 is a repellent for retinal ganglion cell axons. J Neurosci. 2000;20(13):4962–4974. 10864954 10.1523/JNEUROSCI.20-13-04962.2000 PMC6772294

[36] Ruzafa N, Pereiro X, Aspichueta P, Araiz J, Vecino E. The retina of osteopontin deficient mice in aging. Mol Neurobiol. 2018;55(1):213–221. 28866734 10.1007/s12035-017-0734-9 PMC5808060

[37] Thoreson WB, Mangel SC. Lateral interactions in the outer retina. Prog Retin Eye Res. 2012;31(5):407–441. 22580106 10.1016/j.preteyeres.2012.04.003 PMC3401171

[38] Lu Y, Shiau F, Yi W, Lu S, Wu Q, Pearson JD, Kallman A, Zhong S, Hoang T, Zuo Z, et al. Single-cell analysis of human retina identifies evolutionarily conserved and species-specific mechanisms controlling development. Dev Cell. 2020;53(4):473–491.e9. 32386599 10.1016/j.devcel.2020.04.009 PMC8015270

[39] Kay JN, Chu MW, Sanes JR. MEGF10 and MEGF11 mediate homotypic interactions required for mosaic spacing of retinal neurons. Nature. 2012;483(7390):465–469. 22407321 10.1038/nature10877 PMC3310952

[40] Winslow RL, Miller RF, Ogden TE. Functional role of spines in the retinal horizontal cell network. Proc Natl Acad Sci USA. 1989;86(1):387–391. 2463626 10.1073/pnas.86.1.387 PMC286470

[41] Hirano AA, Vuong HE, Kornmann HL, Schietroma C, Stella SL Jr, Barnes S, Brecha NC. Vesicular release of GABA by mammalian horizontal cells mediates inhibitory output to photoreceptors. Front Cell Neurosci. 2020;14: Article 600777. 33335476 10.3389/fncel.2020.600777 PMC7735995

[42] Behrens C, Yadav SC, Korympidou MM, Zhang Y, Haverkamp S, Irsen S, Schaedler A, Lu X, Liu Z, Lause J, et al. Retinal horizontal cells use different synaptic sites for global feedforward and local feedback signaling. Curr Biol. 2022;32(3):545–558.e5. 34910950 10.1016/j.cub.2021.11.055 PMC8886496

[43] Provis JM. Development of the primate retinal vasculature. Prog Retin Eye Res. 2001;20(6):799–821. 11587918 10.1016/s1350-9462(01)00012-x

[44] Hahn J, Monavarfeshani A, Qiao M, Kao AH, Kölsch Y, Kumar A, Kunze VP, Rasys AM, Richardson R, Wekselblatt JB, et al. Evolution of neuronal cell classes and types in the vertebrate retina. Nature. 2023;624(7991):415–424. 38092908 10.1038/s41586-023-06638-9 PMC10719112

[45] Kay P, Yang YC, Hiscott P, Gray D, Maminishkis A, Paraoan L. Age-related changes of cystatin C expression and polarized secretion by retinal pigment epithelium: Potential age-related macular degeneration links. Invest Ophthalmol Vis Sci. 2014;55(2):926–934. 24458156 10.1167/iovs.13-13239 PMC11980428

[46] Yalaz C, Bridges E, Alham NK, Zois CE, Chen J, Bensaad K, Miar A, Pires E, Muschel RJ, McCullagh JSO, et al. Cone photoreceptor phosphodiesterase PDE6H inhibition regulates cancer cell growth and metabolism, replicating the dark retina response. Cancer Metab. 2024;12(1):5. 38350962 10.1186/s40170-023-00326-y PMC10863171

[47] Huang H. Pericyte-endothelial interactions in the retinal microvasculature. Int J Mol Sci. 2020;21(19):7413. 33049983 10.3390/ijms21197413 PMC7582747

[48] Buldyrev I, Puthussery T, Taylor WR. Synaptic pathways that shape the excitatory drive in an OFF retinal ganglion cell. J Neurophysiol. 2012;107(7):1795–1807. 22205648 10.1152/jn.00924.2011 PMC3331668

[49] DeVries SH, Schwartz EA. Kainate receptors mediate synaptic transmission between cones and ‘Off’ bipolar cells in a mammalian retina. Nature. 1999;397(6715):157–160. 9923677 10.1038/16462

[50] Dong LD, Gao F, Wang XH, Miao Y, Wang SY, Wu Y, Li F, Wu J, Cheng XL, Sun XH, et al. GluA2 trafficking is involved in apoptosis of retinal ganglion cells induced by activation of EphB/EphrinB reverse signaling in a rat chronic ocular hypertension model. J Neurosci. 2015;35(13):5409–5421. 25834064 10.1523/JNEUROSCI.4376-14.2015 PMC6705403

[51] KhalafAllah MT, Fuchs PA, Nugen F, El Hamdaoui M, Levy A, Redden DT, Samuels BC, Grytz R. Longitudinal changes of Bruch’s membrane opening, anterior scleral canal opening, and border tissue in experimental juvenile high myopia. Invest Ophthalmol Vis Sci. 2023;64(4):2. 10.1167/iovs.64.4.2 PMC10080949 37010856

[52] Sajdak BS, Salmon AE, Cava JA, Allen KP, Freling S, Ramamirtham R, Norton TT, Roorda A, Carroll J. Noninvasive imaging of the tree shrew eye: Wavefront analysis and retinal imaging with correlative histology. Exp Eye Res. 2019;185: Article 107683. 31158381 10.1016/j.exer.2019.05.023 PMC6698412

[53] Samuels BC, Siegwart JT, Zhan W, Hethcox L, Chimento M, Whitley R, Downs JC, Girkin CA. A novel tree shrew (Tupaia belangeri) model of glaucoma. Invest Ophthalmol Vis Sci. 2018;59(7):3136–3143. 30025140 10.1167/iovs.18-24261 PMC6018453

[54] Blasiak J, Sobczuk P, Pawlowska E, Kaarniranta K. Interplay between aging and other factors of the pathogenesis of age-related macular degeneration. Ageing Res Rev. 2022;81: Article 101735. 36113764 10.1016/j.arr.2022.101735

[55] Buhr ED. Tangled up in blue: Contribution of short-wavelength sensitive cones in human circadian photoentrainment. Proc Natl Acad Sci USA. 2023;120(2): Article e2219617120. 36598954 10.1073/pnas.2219617120 PMC9926240

[56] Fitzpatrick MJ, Kerschensteiner D. Homeostatic plasticity in the retina. Prog Retin Eye Res. 2023;94: Article 101131. 36244950 10.1016/j.preteyeres.2022.101131

[57] Sanes JR, Masland RH. The types of retinal ganglion cells: Current status and implications for neuronal classification. Annu Rev Neurosci. 2015;38:221–246. 25897874 10.1146/annurev-neuro-071714-034120

[58] Peichl L, Kaiser A, Rakotondraparany F, Dubielzig RR, Goodman SM, Kappeler PM. Diversity of photoreceptor arrangements in nocturnal, cathemeral and diurnal Malagasy lemurs. J Comp Neurol. 2019;527(1):13–37. 28054342 10.1002/cne.24167

[59] Vargas A, Kim HS, Baral E, Yu WQ, Craft CM, Lee EJ. Protective effect of clusterin on rod photoreceptor in rat model of retinitis pigmentosa. PLOS ONE. 2017;12(8): Article e0182389. 28767729 10.1371/journal.pone.0182389 PMC5540409

[60] Zhang H, Kim JK, Edwards CA, Xu Z, Taichman R, Wang CY. Clusterin inhibits apoptosis by interacting with activated Bax. Nat Cell Biol. 2005;7(9):909–915. 16113678 10.1038/ncb1291

[61] Quesada A, Prada FA, Genis-Galvez JM. Bipolar cells in the chicken retina. J Morphol. 1988;197(3):337–351. 29874893 10.1002/jmor.1051970308

[62] Shekhar K, Lapan SW, Whitney IE, Tran NM, Macosko EZ, Kowalczyk M, Adiconis X, Levin JZ, Nemesh J, Goldman M, et al. Comprehensive classification of retinal bipolar neurons by single-cell transcriptomics. Cell. 2016;166(5):1308–1323.e30. 27565351 10.1016/j.cell.2016.07.054 PMC5003425

[63] Fazzari P, Paternain AV, Valiente M, Pla R, Luján R, Lloyd K, Lerma J, Marín O, Rico B. Control of cortical GABA circuitry development by Nrg1 and ErbB4 signalling. Nature. 2010;464:1376–1380. 20393464 10.1038/nature08928

[64] Lu Y, Sun XD, Hou FQ, Bi LL, Yin DM, Liu F, Chen YJ, Bean JC, Jiao HF, Liu X, et al. Maintenance of GABAergic activity by neuregulin 1-ErbB4 in amygdala for fear memory. Neuron. 2014;84(4):835–846. 25451196 10.1016/j.neuron.2014.09.029

[65] Shamir A, Kwon OB, Karavanova I, Vullhorst D, Leiva-Salcedo E, Janssen MJ, Buonanno A. The importance of the NRG-1/ErbB4 pathway for synaptic plasticity and behaviors associated with psychiatric disorders. J Neurosci. 2012;32(9):2988–2997. 22378872 10.1523/JNEUROSCI.1899-11.2012 PMC3302154

[66] Yan W, Laboulaye MA, Tran NM, Whitney IE, Benhar I, Sanes JR. Mouse retinal cell atlas: Molecular identification of over sixty amacrine cell types. J Neurosci. 2020;40(27):5177–5195. 32457074 10.1523/JNEUROSCI.0471-20.2020 PMC7329304

[67] Menger N, Pow DV, Wassle H. Glycinergic amacrine cells of the rat retina. J Comp Neurol. 1998;401(1):34–46. 9802699 10.1002/(sici)1096-9861(19981109)401:1<34::aid-cne3>3.0.co;2-p

[68] Weiner GA, Shah SH, Angelopoulos CM, Bartakova AB, Pulido RS, Murphy A, Nudleman E, Daneman R, Goldberg JL. Cholinergic neural activity directs retinal layer-specific angiogenesis and blood retinal barrier formation. Nat Commun. 2019;10(1):2477. 31171770 10.1038/s41467-019-10219-8 PMC6554348

[69] Taylor WR, Smith RG. The role of starburst amacrine cells in visual signal processing. Vis Neurosci. 2012;29(1):73–81. 22310373 10.1017/S0952523811000393 PMC3292856

[70] Hisaoka T, Nakamura Y, Senba E, Morikawa Y. The forkhead transcription factors, Foxp1 and Foxp2, identify different subpopulations of projection neurons in the mouse cerebral cortex. Neuroscience. 2010;166(2):551–563. 20040367 10.1016/j.neuroscience.2009.12.055

[71] Fisher SE, Scharff C. FOXP2 as a molecular window into speech and language. Trends Genet. 2009;25(4):166–177. 19304338 10.1016/j.tig.2009.03.002

[72] Rousso DL, Qiao M, Kagan RD, Yamagata M, Palmiter RD, Sanes JR. Two pairs of ON and OFF retinal ganglion cells are defined by intersectional patterns of transcription factor expression. Cell Rep. 2016;15(9):1930–1944. 27210758 10.1016/j.celrep.2016.04.069 PMC4889540

[73] Casson RJ, Chidlow G, Crowston JG, Williams PA, Wood JPM. Retinal energy metabolism in health and glaucoma. Prog Retin Eye Res. 2021;81: Article 100881. 32712136 10.1016/j.preteyeres.2020.100881

[74] Duan X, Qiao M, Bei F, Kim IJ, He Z, Sanes JR. Subtype-specific regeneration of retinal ganglion cells following axotomy: Effects of osteopontin and mTOR signaling. Neuron. 2015;85(6):1244–1256. 25754821 10.1016/j.neuron.2015.02.017 PMC4391013

[75] Peichl L, Ott H, Boycott BB. Alpha ganglion cells in mammalian retinae. Proc R Soc Lond B Biol Sci. 1987;231(1263):169–197. 2889210 10.1098/rspb.1987.0040

[76] Tapia ML, Nascimento-Dos-Santos G, Park KK. Subtype-specific survival and regeneration of retinal ganglion cells in response to injury. Front Cell Dev Biol. 2022;10: Article 956279. 36035999 10.3389/fcell.2022.956279 PMC9411869

[77] Peichl L, Gonzalez-Soriano J. Morphological types of horizontal cell in rodent retinae: A comparison of rat, mouse, gerbil, and guinea pig. Vis Neurosci. 1994;11(3):501–517. 8038125 10.1017/s095252380000242x

[78] Dacey DM, Crook JD, Packer OS. Distinct synaptic mechanisms create parallel S-ON and S-OFF color opponent pathways in the primate retina. Vis Neurosci. 2014;31(2):139–151. 23895762 10.1017/S0952523813000230 PMC4309572

[79] Fischer MD, Michalakis S, Wilhelm B, Zobor D, Muehlfriedel R, Kohl S, Weisschuh N, Ochakovski GA, Klein R, Schoen C, et al. Safety and vision outcomes of subretinal gene therapy targeting cone photoreceptors in Achromatopsia: A nonrandomized controlled trial. JAMA Ophthalmol. 2020;138(6):643–651. 32352493 10.1001/jamaophthalmol.2020.1032 PMC7193523

[80] Jin S, Guerrero-Juarez CF, Zhang L, Chang I, Ramos R, Kuan CH, Myung P, Plikus MV, Nie Q. Inference and analysis of cell-cell communication using CellChat. Nat Commun. 2021;12(1):1088. 33597522 10.1038/s41467-021-21246-9 PMC7889871

[81] Liu ST, Zhong SM, Li XY, Gao F, Li F, Zhang ML, Zhu K, Sun XH, Wang X, Miao Y, et al. EphrinB/EphB forward signaling in Müller cells causes apoptosis of retinal ganglion cells by increasing tumor necrosis factor alpha production in rat experimental glaucomatous model. Acta Neuropathol Commun. 2018;6(1):111. 30355282 10.1186/s40478-018-0618-x PMC6201539

[82] McGinnis CS, Murrow LM, Gartner ZJ. DoubletFinder: Doublet detection in single-cell RNA sequencing data using artificial nearest neighbors. Cell Syst. 2019;8(4):329–337.e4. 30954475 10.1016/j.cels.2019.03.003 PMC6853612

[83] Zhang H, Li J, Ren J, Sun S, Ma S, Zhang W, Yu Y, Cai Y, Yan K, Li W, et al. Single-nucleus transcriptomic landscape of primate hippocampal aging. Protein Cell. 2021;12(9):695–716. 34052996 10.1007/s13238-021-00852-9 PMC8403220

[84] Stuart T, Butler A, Hoffman P, Hafemeister C, Papalexi E, Mauck WM III, Hao Y, Stoeckius M, Smibert P, Satija R. Comprehensive integration of single-cell data. Cell. 2019;177:1888–1902.e21. 31178118 10.1016/j.cell.2019.05.031 PMC6687398

[85] Schirmer L, Velmeshev D, Holmqvist S, Kaufmann M, Werneburg S, Jung D, Vistnes S, Stockley JH, Young A, Steindel M, et al. Neuronal vulnerability and multilineage diversity in multiple sclerosis. Nature. 2019;573(7772):75–82. 31316211 10.1038/s41586-019-1404-z PMC6731122

[86] Yu G, Wang LG, Han Y, He QY. clusterProfiler: An R package for comparing biological themes among gene clusters. OMICS. 2012;16(5):284–287. 22455463 10.1089/omi.2011.0118 PMC3339379

[87] Butler A, Hoffman P, Smibert P, Papalexi E, Satija R. Integrating single-cell transcriptomic data across different conditions, technologies, and species. Nat Biotechnol. 2018;36(5):411–420. 29608179 10.1038/nbt.4096 PMC6700744

[88] Schwartz GW, Zhou Y, Petrovic J, Fasolino M, Xu L, Shaffer SM, Pear WS, Vahedi G, Faryabi RB. TooManyCells identifies and visualizes relationships of single-cell clades. Nat Methods. 2020;17:405–413. 32123397 10.1038/s41592-020-0748-5 PMC7439807

[89] La Manno G, Soldatov R, Zeisel A, Braun E, Hochgerner H, Petukhov V, Lidschreiber K, Kastriti ME, Lönnerberg P, Furlan A, et al. RNA velocity of single cells. Nature. 2018;560(7719):494–498. 30089906 10.1038/s41586-018-0414-6 PMC6130801

[90] Bergen V, Lange M, Peidli S, Wolf FA, Theis FJ. Generalizing RNA velocity to transient cell states through dynamical modeling. Nat Biotechnol. 2020;38(12):1408–1414. 32747759 10.1038/s41587-020-0591-3

[91] Aibar S, González-Blas CB, Moerman T, Huynh-Thu VA, Imrichova H, Hulselmans G, Rambow F, Marine JC, Geurts P, Aerts J, et al. SCENIC: Single-cell regulatory network inference and clustering. Nat Methods. 2017;14(11):1083–1086. 28991892 10.1038/nmeth.4463 PMC5937676

