# Supplementary material for: Cellular Characterization and Interspecies Evolution of the Tree Shrew Retina across Postnatal Lifespan
Source: Research (Wash D C). 2024 Nov 21;7:0536. doi: 10.34133/research.0536 (PMC11579486; doi:10.34133/research.0536)
Supplement: Supplementary 1 — Figs. S1 to S10 Tables S1 to S12 [file research.0536.f1.zip › Supplementary materials.docx]

**Supplementary figures 1-9**

**
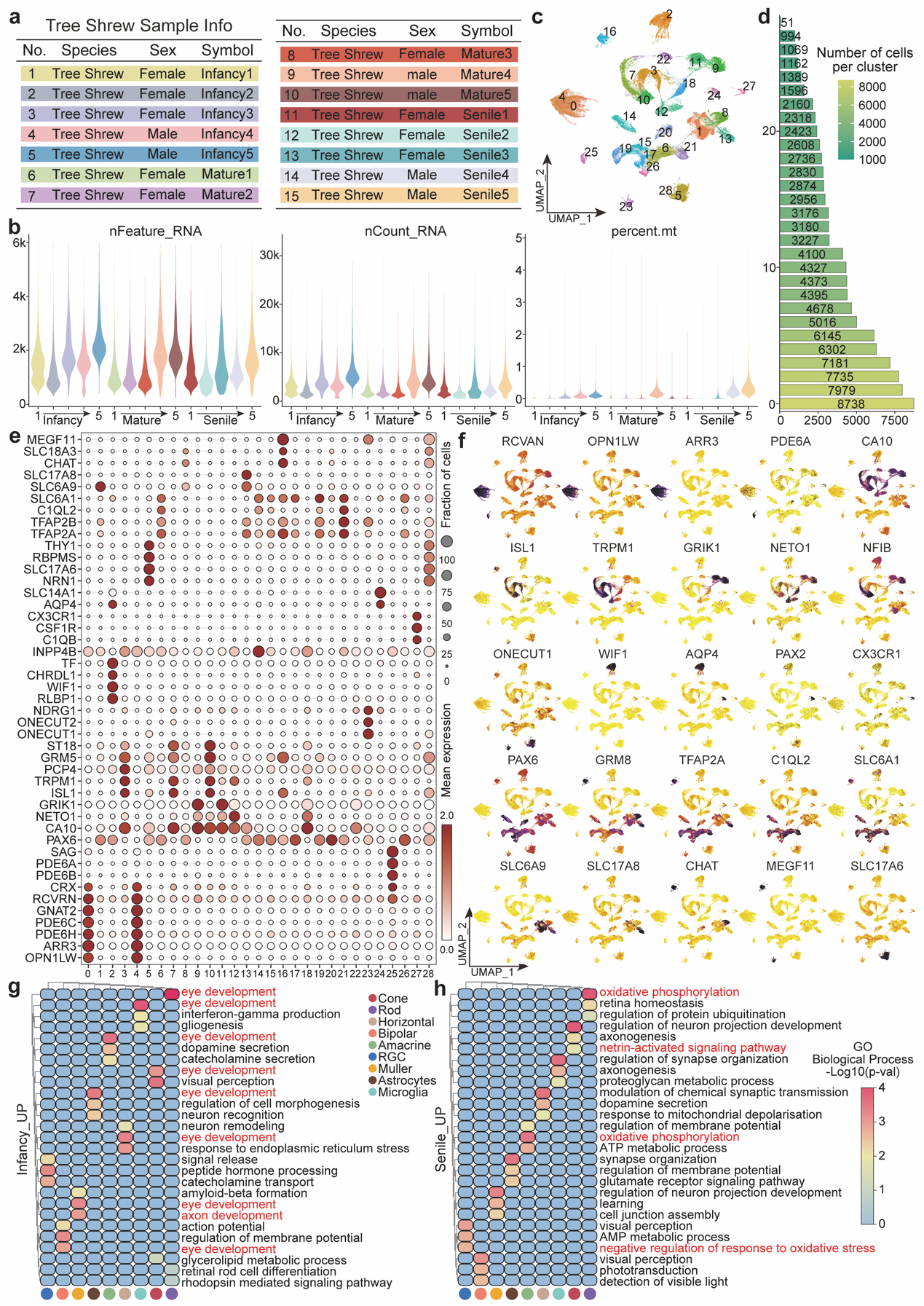
**

**Supplementary Fig. 1 Cellular composition of TS retinas by snRNA-seq analysis. a** Detailed information of individual TS included in this study. **b** Violin plots show 15 snRNA-seq samples and data quality of nFeature_RNA, nCount_RNA and percent.mt. **c** UMAP depicts the distribution of 29 cell clusters. **d** The bar chart shows the cell number of 29 clusters. **e** The dotplot depicts the expression levels of specific markers across 29 clusters. **f** The UMAP shows the expression of specific markers of 9 cell types. **g, h** The representative GO terms enriched by upregulated DEGs of 9 retinal cell populations in the infant and senile groups. UMAP, uniform manifold and projection.

**
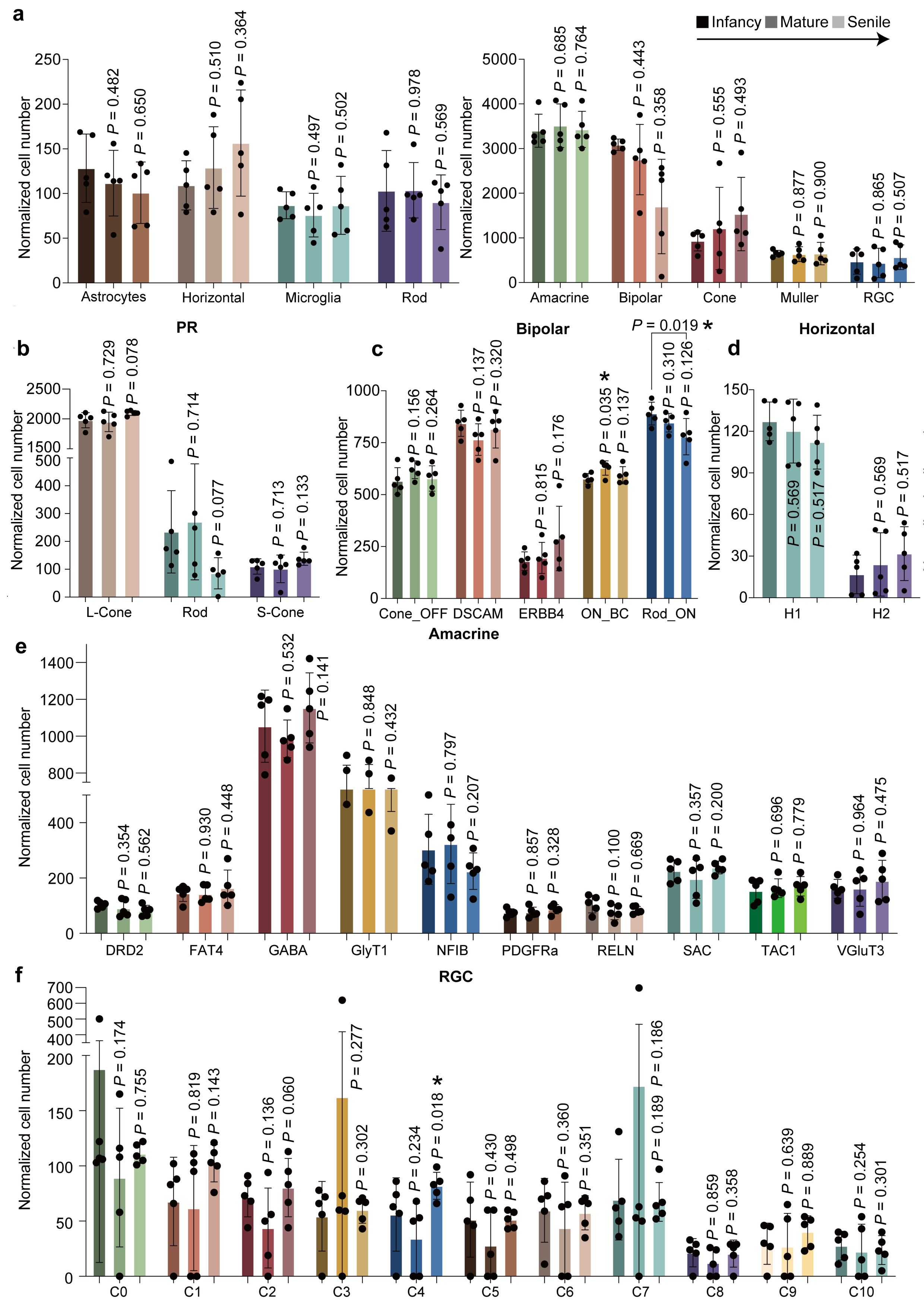
**

**Supplementary Fig. 2 The age-related changes of normalized cell number per clusters in TS retina.** **a** Bar charts of the normalized cell number of 9 retinal cell populations grouped by age. **b-f** Bar charts of the normalized cell number of retinal cell subpopulations grouped by age. *n* = 5 per group. **P* < 0.05 by one-way ANOVA. Error bars, standard error of mean. PR, photoreceptor cells; RGC, retinal ganglion cell; SAC, starburst amacrine cell; BC, bipolar cell; L-Cone, longwave-sensitive cone; S-Cone, shortwave-sensitive cone cell.

**
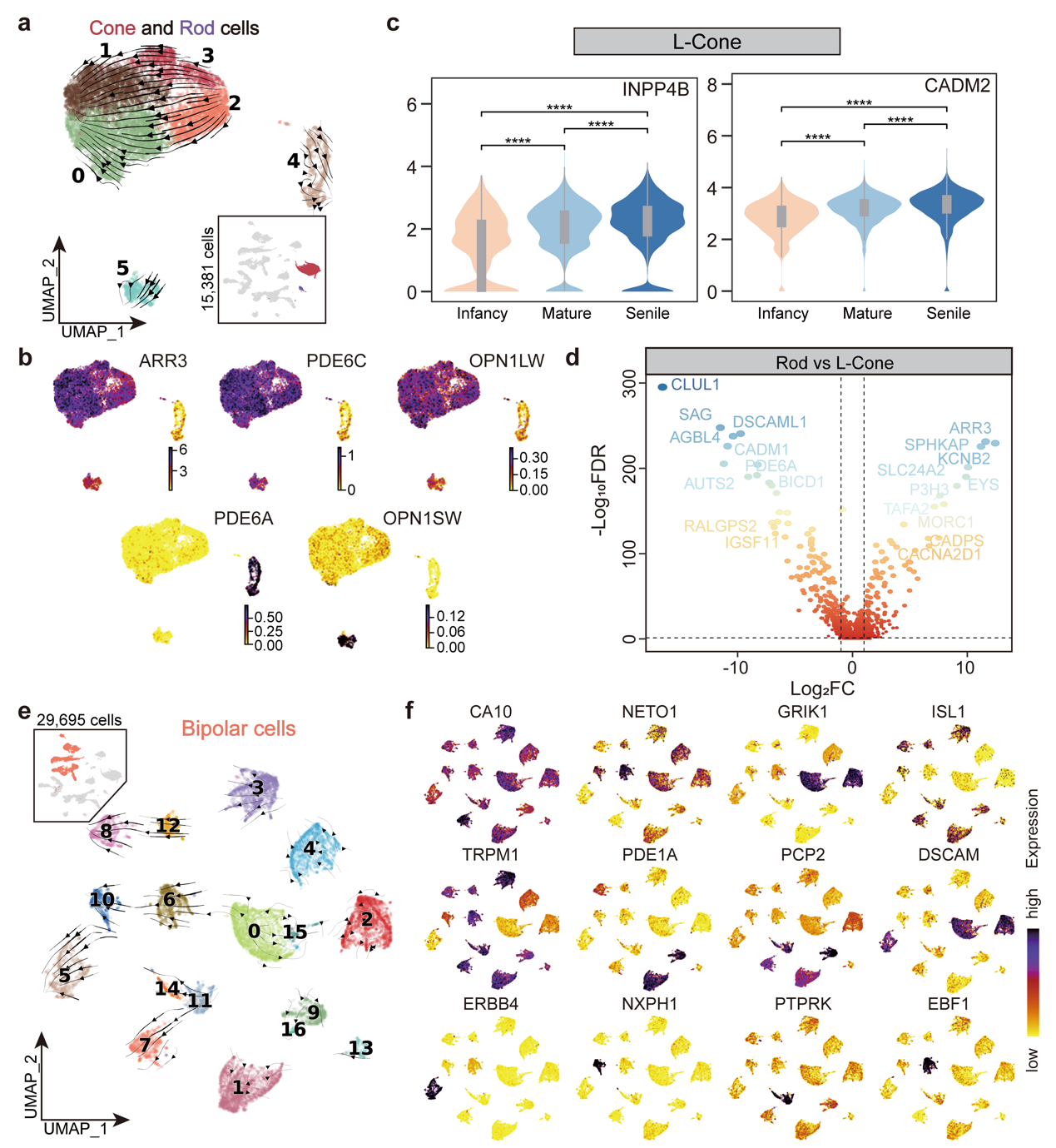
**

**Supplementary Fig. 3 Characteristics of photoreceptors and BCs**

**a** The subclusters of photoreceptor cells are visualized by UMAP. **b** The expression of representative marker genes of photoreceptor subtypes. **c** The violin diagram presents the expression of *INPP4B* and *CADM2* in the L-cones the infant, mature, and senile groups. **d** The top 10 DEGs in rods and L-cones displayed in volcano plot. **e** Clustering of BCs subclasses depicted in UMAP. **f** The expression of the selected markers in BCs subclasses. UMAP, uniform manifold and projection; Log_2_FC, Log_2_ fold change. *****P* < 0.0001.


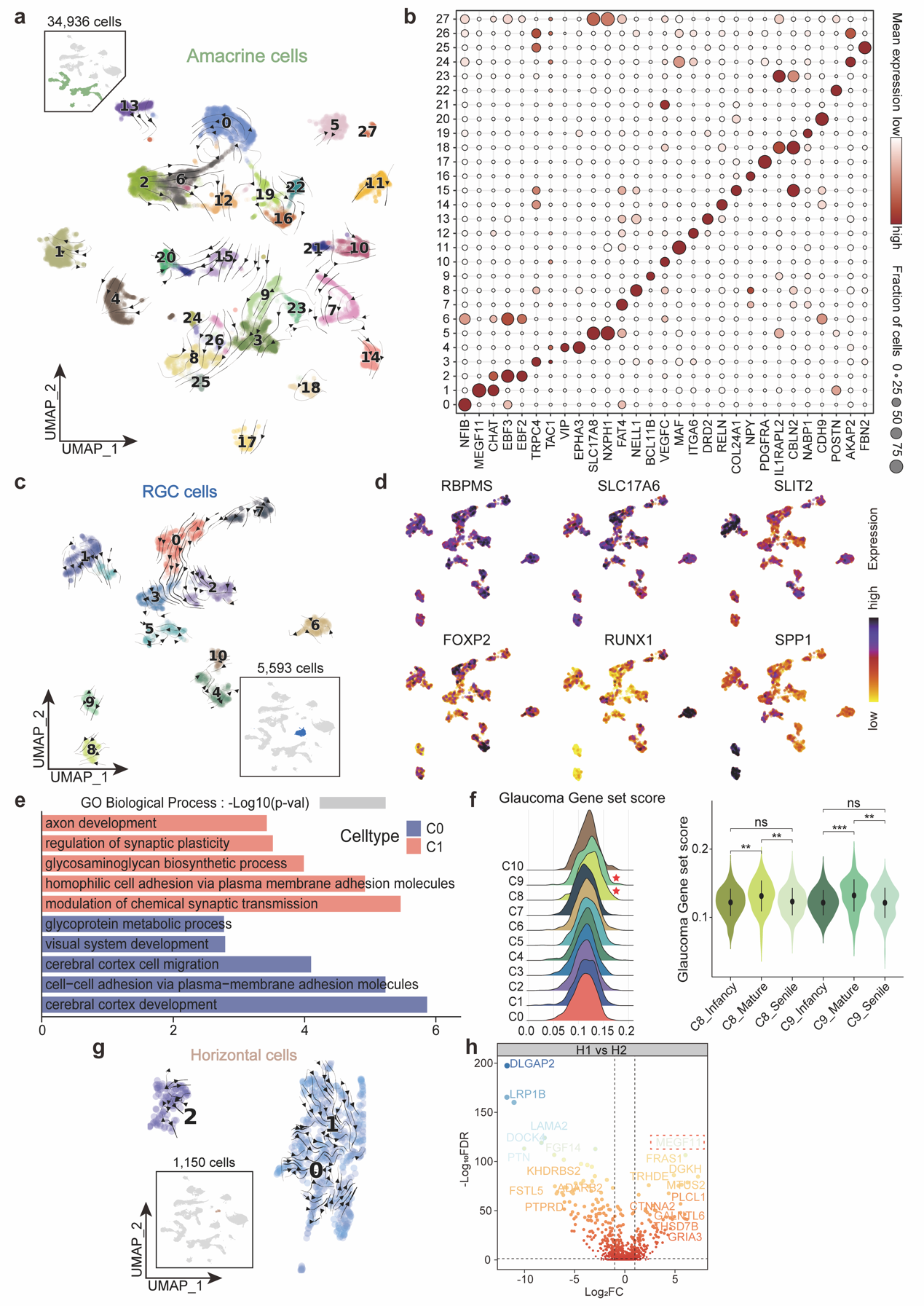


**Supplementary Fig. 4 Identification of ACs, RGCs, HCs and their subtypes. a** Clustering of ACs subpopulations viewed by UMAP. **b** The dotplot shows the expression of selected genes expressed in subsets of ACs. The size of the dot represents the percentage of cells expressed by the selected genes in a cluster. The degree of color refers to the average expression of genes within cells. **c** UMAP depicts RGCs subclusters. **d** The expression of the selected markers in RGCs subclasses. **e** Annotation of enrichment analysis of DEGs in C1 and C0, respectively. The horizontal coordinator is -log10 (*p*-val). **f** The disease gene set scores display the association of RGCs subclusters with Glaucoma at different age stages. The red five-pointed stars represent C8, C9. **g** Clustering of HCs subclasses viewed by UMAP. **h** The top 10 DEGs in H1 and H2 displayed in volcano plot. The red dashed box represents the most upregulated DEG in H1 as compared to H2. UMAP, uniform manifold and projection; RGC, retinal ganglion cell; Log_2_FC, Log_2_fold change; ns, no significance. ^**^*P* ≤ 0.01, ^***^*P* ≤ 0.001.

**
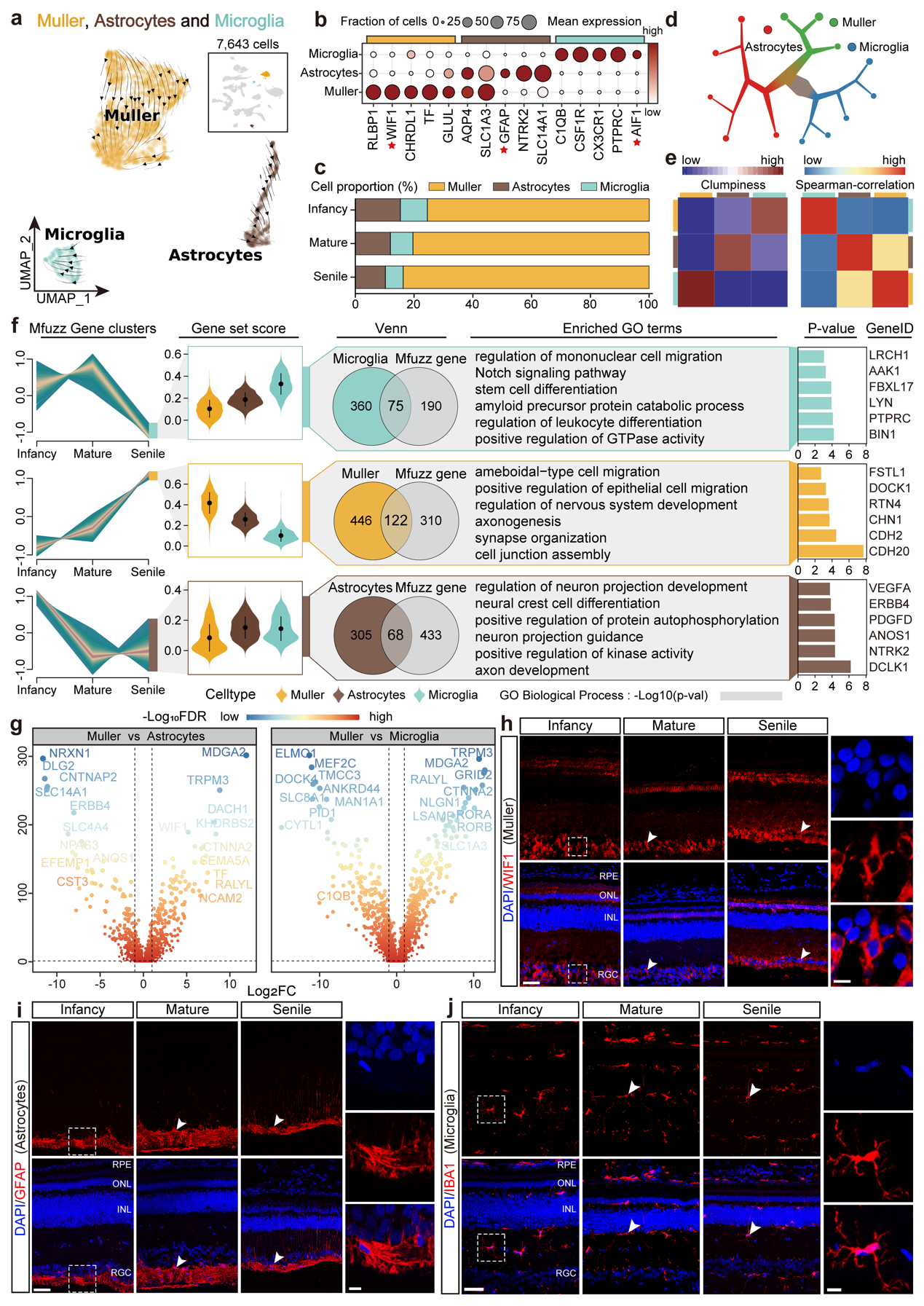
**

**Supplementary Fig. 5 Features of Müller, astrocytes and microglia. a** Clustering of glial cells subclusters visualized by UMAP. **b, c** The expression of representative marker genes in glial subpopulations and cell proportions of glial cells. The red five-pointed stars represent important marker genes. **d** TooManyCells tree shows the evolutionary relationship among retinal glial cells. **e** Heatmap shows clumpiness and correlation of 3 glial cell types. Clumpiness: Parameters to quantify the degree of aggregation or colocalization of different cell populations. **f** Mfuzz plot clusters the downregulated genes or upregulated genes in the glial cells with age. Associations of these downregulated genes or upregulated genes with down-DEGs or up-DEGs in 3 subclasses are revealed by gene set scoring, respectively. Venn plot of age-related downregulated genes or upregulated genes in glial cells with down-DEGs in microglia, with up-DEGs in Müller, with down-DEGs in astrocytes, respectively (adjusted *P*-value < 0.05, log_2_FC > 0.25). The GO terms enriched by the overlapped up-DEGs or down-DEGs in Müller, microglia, and astrocytes. **g** The top 10 DEGs in Müller, microglia and astrocytes displayed in volcano plot. **h-j** Immunostaining identification of Müller (*WIF1*^+^, red, h), astrocytes (*GFAP*^+^, red, i) and microglia (*IBA1*^+^, red, j) location in the TS retina in the infant, mature and senile groups. Blue, DAPI. Scale bar: low magnification, 50 μm; high magnification, 10 μm. *n* = 3 TS per group. White arrows indicate the positive immunostaining, and the white dashed boxes represent the zoom area of the positive immunostaining. UMAP, uniform manifold and projection; Log_2_FC, Log_2_fold change. RPE, retinal pigment epithelium; ONL, outer nuclear layer; INL, inner nuclear layer; RGC, retinal ganglion cell layer; GO, gene ontology.


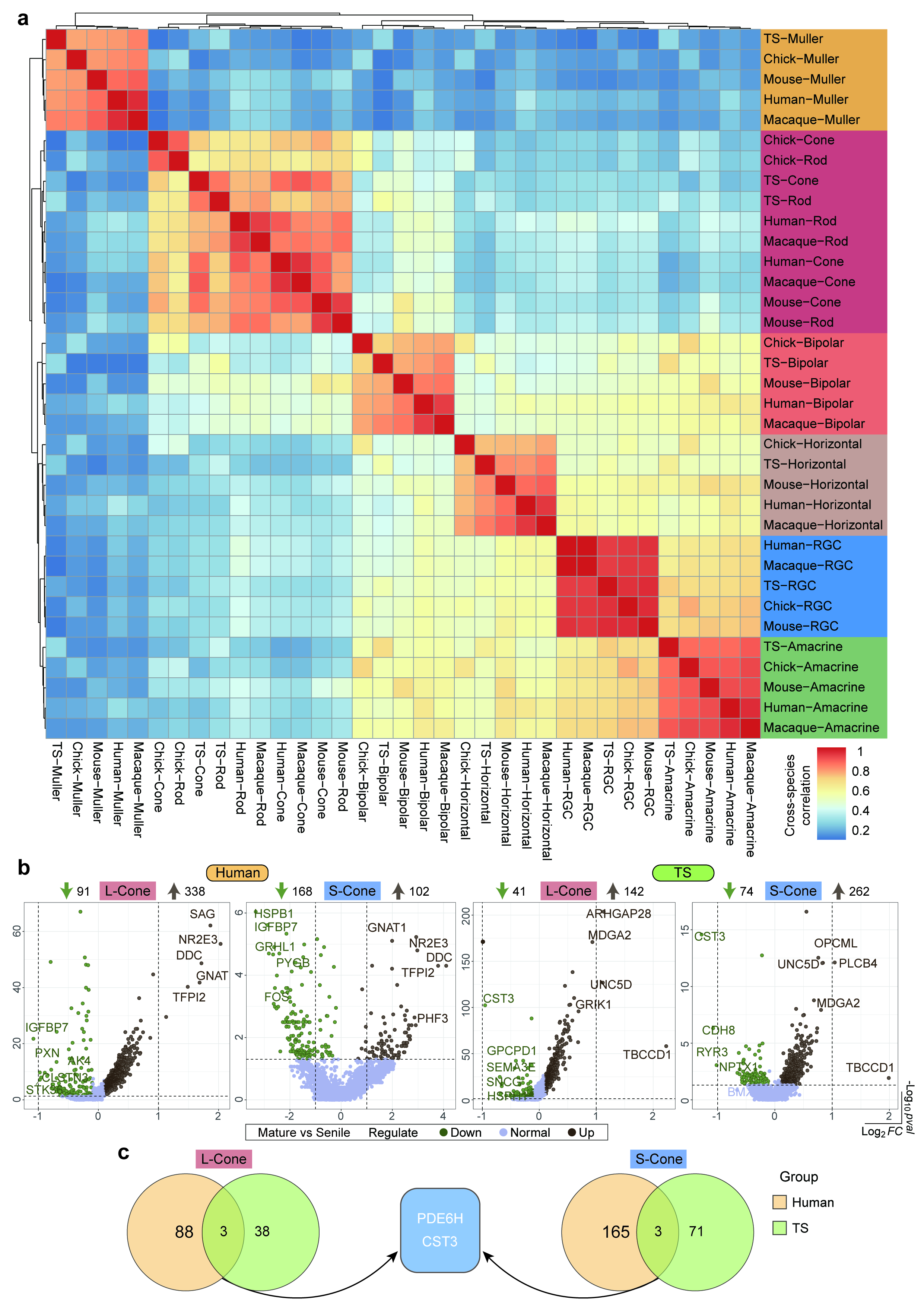


**Supplementary Fig. 6 Cross-species comparison of retinal cellular transcriptomes and gene expression regulation of cone subtypes. a** The heat map shows transcriptional correlation of each cell subpopulation across species. **b** Comparison of gene expression differences in different cone cell subtypes (L-Cone and S-Cone) between humans and TS, particularly the changes between mature and senile stages. **c** The Venn diagram shows the shared and unique gene expression in L-Cone and S-Cone between humans and TS. Each circle represents a species, with the overlapping area indicating the shared genes between the two species. RGC: retinal ganglion cell; TS: tree shrews.


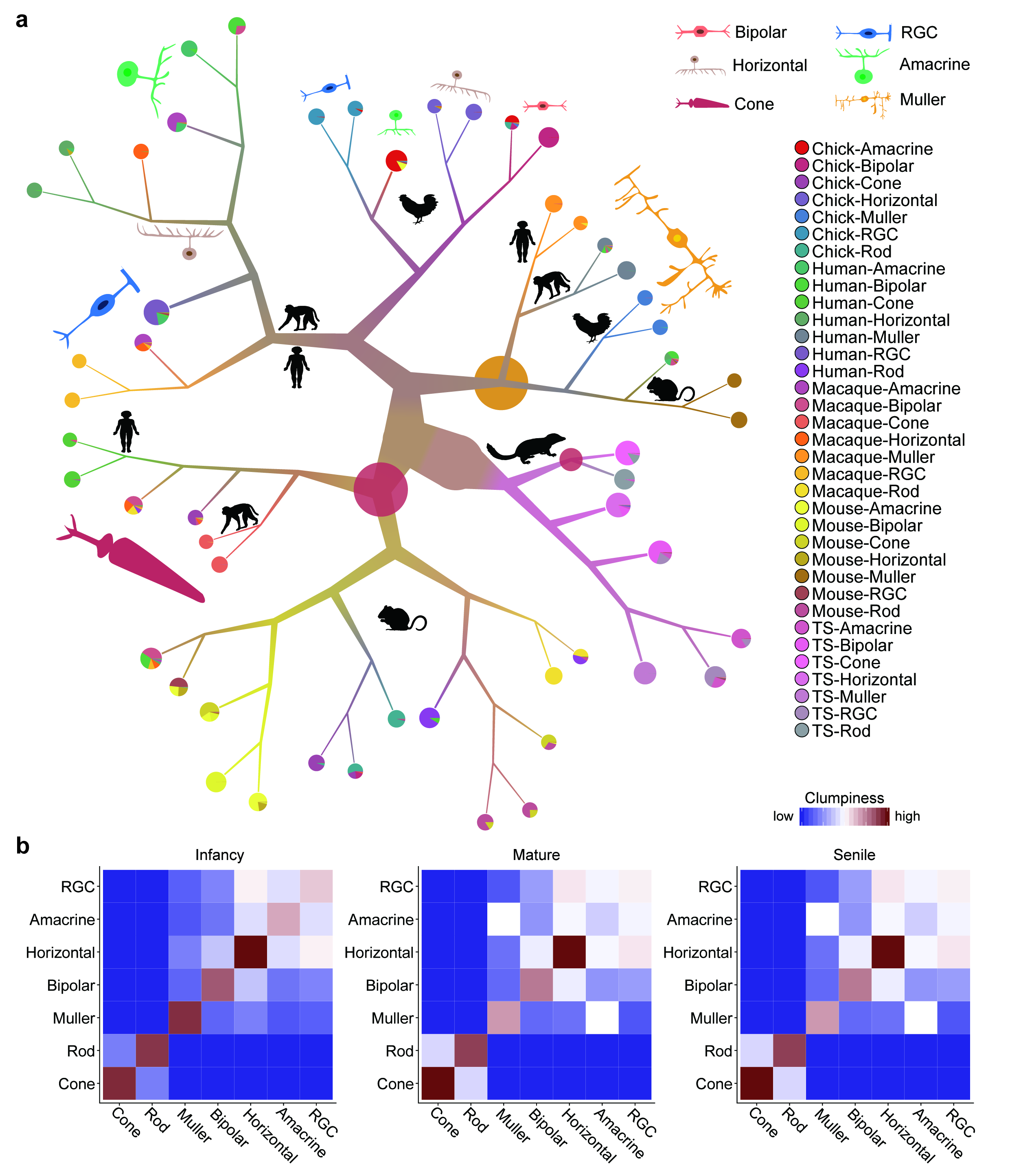


**Supplementary Fig. 7 Cross-species comparison of retinal cellular transcriptomes across different ages. a** TooManyCells tree shows relationships of single-cell clades across humans, macaques, mice, chicks, and TS retinal cell populations. **b** Heatmap shows clumpiness of the different cell types in the infant, mature and senile groups. Clumpiness: Parameters to quantify the degree of aggregation or colocalization of different cell populations. RGC: retinal ganglion cell; TS: tree shrews.


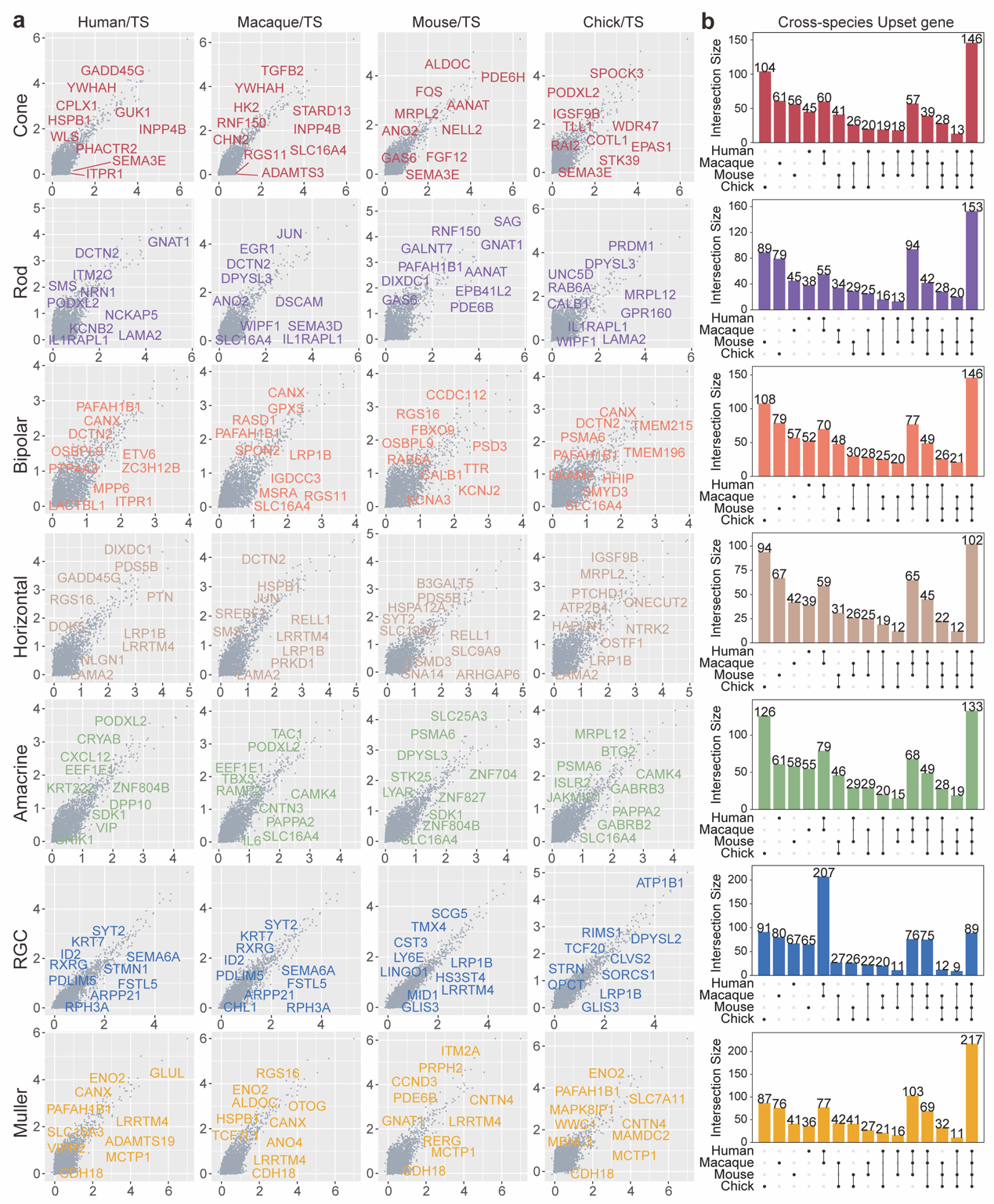


**Supplementary Fig. 8 Retinal single-cell transcriptome differences across different species. a** The volcano map shows the DEGs of different cell types in the humans, macaques, mice, chicks, and TS. **b** The cross-species cell specific DEGs. The dots on the abscissa represent the indicated species compared to TS. The number on the column chart represent the specific genes of TS as compared to the related species indicated by dots.


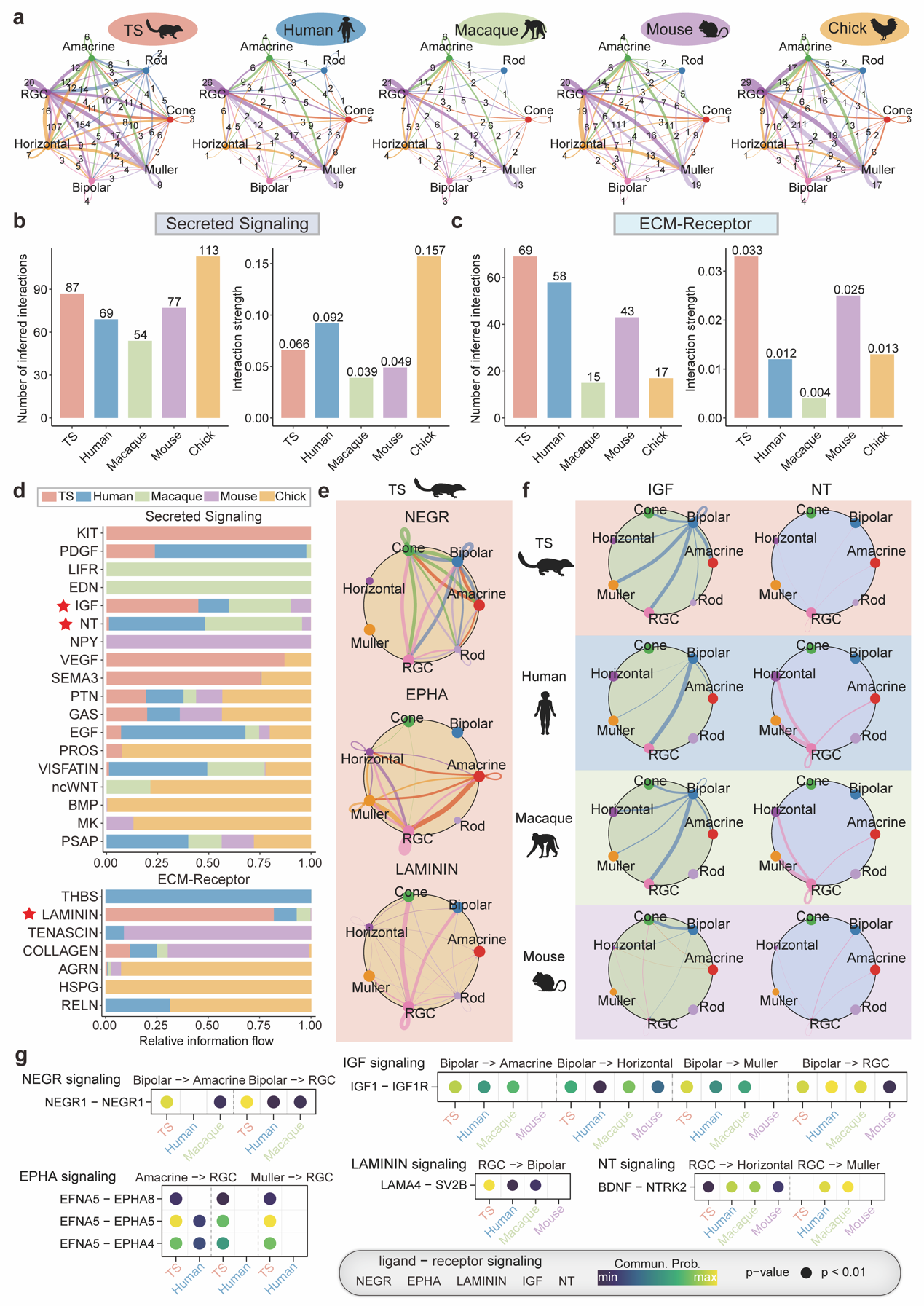


**Supplementary Fig. 9 Cellular communication of retinal cells across species. a** Networks display the intercellular communication of retinal cells across humans, macaques, mice, chicks, and TS. **b, c** Bar charts present the cross-species differences in the number and intensity of Secreted signaling and ECM-Receptor communication means in the retina. **d** The relative information flow of retinal cells by Secreted signaling and ECM-Receptor ways across different species. The red five-pointed stars represent important signalling in the TS retina cells. **e, f** Networks visualize the intercellular communication of retinal cells in *NEGR*, *EPHA*, *LAMININ*, *IGF* and *NT* signaling across humans, macaques, mice, and TS retinas. **g** The intensities of ligand-receptor pairs in the indicated cell types among different species. RGC, retinal ganglion cell; TS, tree shrews.


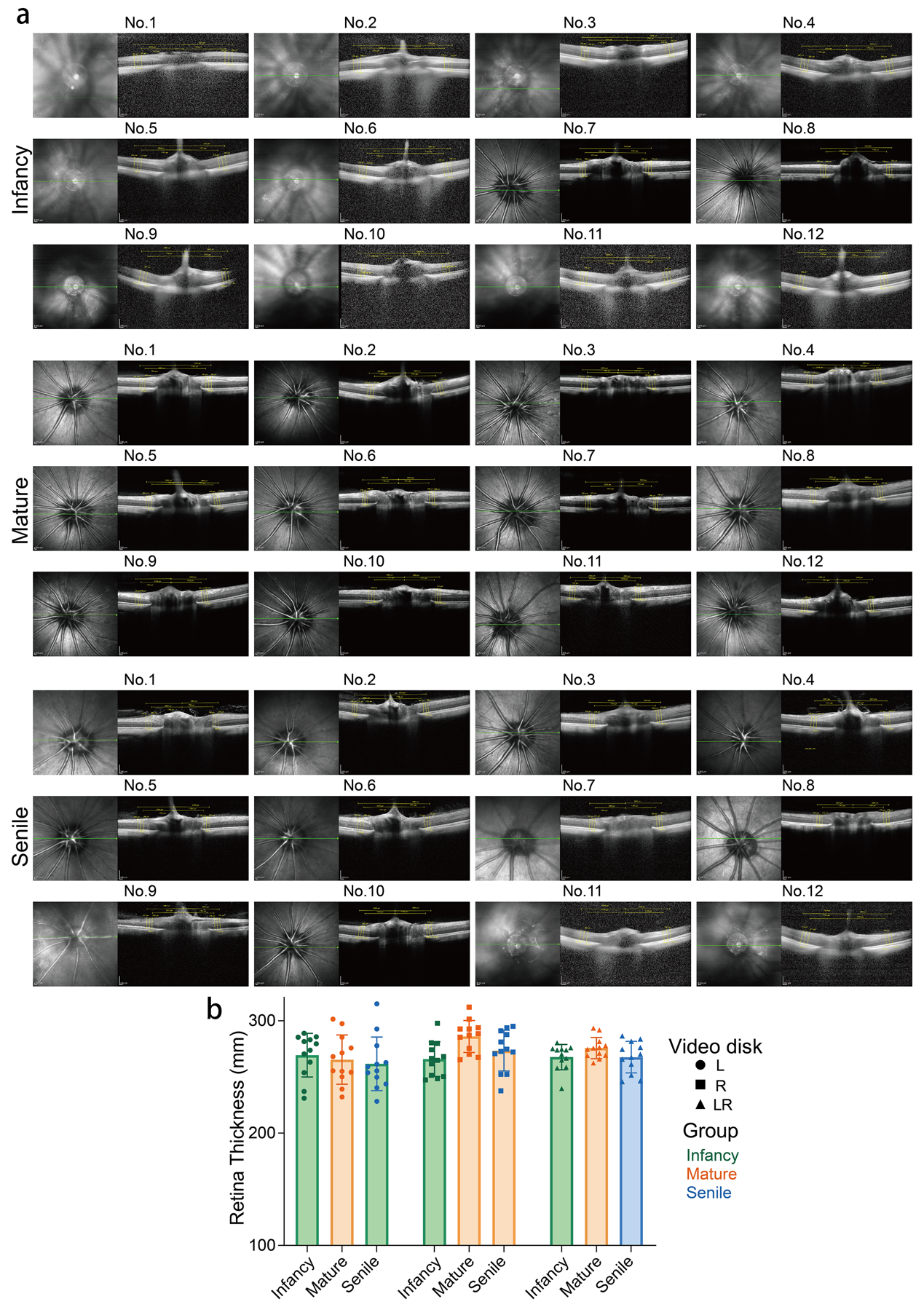


**Supplementary Fig. 10 The thickness of TS retina at different age groups. a** OCT images demonstrate the changes of retinal thickness in the infant, mature and senile TS. **b** Quantification histogram shows the retinal comparison among different age groups. Primary data are shown in the histogram, *n* = 12/group. Data was analyzed by one-way ANOVA. Error bars, standard error of mean. L, left optic disc; R, right optic disc; LR, mean of left optic disc and right optic disc.

**Supplementary Tables 1-12**

**Supplementary Table 1** The overall transcriptomic information and cell populations in the TS retina.

**Supplementary Table 2** Related information of photoreceptor cell subpopulations in the TS retina.

**Supplementary Table 3** Analysis of bipolar cells and their subtypes.

**Supplementary Table 4** Analysis of amacrine cells subtypes in the TS retina.

**Supplementary Table 5** Analysis of retinal ganglion cells subtypes in the TS retina.

**Supplementary Table 6** Analysis of horizontal cells subclasses in the TS retina.

**Supplementary Table 7** Analysis of glial cells subpopulations in the TS retina.

**Supplementary Table 8** Comparative analysis of cross-species transcriptome profiles.

**Supplementary Table 9** Age distribution of TS retina at different stages.

**Supplementary Table 10** Information of integrated snRNA-seq data from TS, humans, macaques, mice and chicks.

**Supplementary Table 11** List of homologous genes of all five species.

**Supplementary Table 12** Antibodies information used in immunostaining experiments.
